# Application of ConvNeXt with transfer learning and data augmentation for malaria parasite detection in resource-limited settings using microscopic images

**DOI:** 10.1371/journal.pone.0313734

**Published:** 2025-06-04

**Authors:** Outlwile Pako Mmileng, Albert Whata, Micheal Olusanya, Siyabonga Mhlongo

**Affiliations:** 1 Centre for Applied Data Science, University of Johannesburg, Johannesburg, South Africa; 2 Department of Statistics, University of Pretoria, Pretoria, South Africa; 3 Department of Computer Science and Information Technology, Sol Plaatje University, Kimberley, South Africa; 4 Department of Applied Information Systems, University of Johannesburg, Johannesburg, South Africa; Prince Mohammad Bin Fahd University, SAUDI ARABIA

## Abstract

Malaria continues to be a severe health problem across the globe, especially within resource-limited areas which lack both skilled diagnostic personnel and diagnostic equipment. This study investigates the use of deep learning diagnosis for malaria through ConvNeXt models that incorporate transfer learning techniques with data augmentation methods for better model performance and transferability. A total number of 606276 thin blood smear images served as the final augmented dataset after the initial 27558 images underwent augmentation. The ConvNeXt Tiny model, version V1 Tiny, achieved an accuracy of 95.9%.; however, the upgraded V2 Tiny Remod version exceeded this benchmark, reaching 98.1% accuracy. The accuracy rate measured 61.4% for Swin Tiny, ResNet18 reached 62.6%, and ResNet50 obtained 81.4%. The combination of label smoothing with the AdamW optimiser produced a model which exhibited strong robustness as well as generalisability. The enhanced ConvNeXt V2 Tiny model combined with data augmentation, transfer learning techniques and explainability frameworks demonstrate a practical solution for malaria diagnosis that achieves high accuracy despite limitations of access to large datasets and microscopy expertise, often observed in resource-limited regions. The findings highlight the potential for real-time diagnostic applications in remote healthcare facilities and the viability of ConvNeXt models in enhancing malaria diagnosis globally.

## 1. Introduction

Malaria is a major global health burden, especially in low and middle-income countries, particularly in sub-Saharan Africa and Northeast Asia [1]. In 2021, the World Health Organization (WHO) estimated 247 million cases and 619,000 deaths from malaria worldwide, with the disease primarily affecting low-income countries [2,3]. Malaria is an infectious disease caused by parasitic protozoa of the genus *plasmodium*, of which *plasmodium falciparum* and *plasmodium vivax* are the most pathogenic to man [4]. These are transmitted through the bites of infected *Anopheles* mosquitoes. Early detection and precise malaria diagnosis play a critical role in lowering both illness severity and death rates, particularly in areas endemic to malaria where healthcare access remains limited [5]. However, access to accurate diagnostic instruments is still problematic, especially in low-income regions [6].

Malaria diagnosis is usually performed by examination of thick and thin giemsa stained blood films where the laboratory technologists manually use microscopes to look for the malaria parasites [7,8]. Even though this approach is reasonably practical, it is rather time-consuming, somewhat subjective and highly dependent on skilled laboratory technicians. Some of the challenges associated with laboratory diagnosis of malaria in low-resource countries include lack of trained personnel, reliance on the skill of the operator and the quality of reagents, while

Polymerase chain reaction (PCR) methods provide high sensitivity and specificity; they are not commonly available in low-resource settings due to their cost, required technical expertise, and cost of diagnostic tests [9]. Using artificial intelligence, specifically deep learning, to overcome these challenges by developing automated diagnostic tools can be an excellent solution to increase diagnostic efficiency and decrease the workload of healthcare professionals in these limited resource settings [10]. However, although promising, the current automated diagnostic tools require substantial computational resources, which may not be feasible in settings with limited hardware availability.

Convolutional neural networks (CNNs), a type of deep learning, are very effective in the automated diagnosis of medical images. CNNs have been applied in many applications, including disease diagnosis, object identification, and segmentation [11]. However, standard CNNs are data-hungry and labelled medical data are limited in many regions worldwide [12]. To address these problems, this study uses the ConvNeXt architecture, which combines the ease of use of conventional CNNs with the hierarchical feature extraction of the latest models, such as vision transformers (ViTs). The research question focuses on how ConvNeXt models can improve diagnostic outcomes in resource-limited settings.

A 16-layer CNN model proposed by [13] identified malaria in thin blood smear images and reached an impressive average accuracy rate of 97.37% during ten-fold cross-validation of 27,578 single-cell images. The research found that CNN models surpassed transfer learning approaches which reached 91.99% accuracy. The CNN model showed strong detection performance because of its superior sensitivity (96.99% versus 89.00%), specificity (97.75% versus 94.98%), and Matthews correlation coefficient scores (94.75% versus 85.25%). Although the study delivered strong results it only performed single-cell classification which restricts its function within wider clinical diagnostic practices.

[14] advanced this effort by introducing a CNN-based system for malaria detection that utilised three different optimisers: Stochastic Gradient Descent (SGD), RMSprop, and Adaptive Moment Estimation (ADAM). The model attained a 96.62% test accuracy with the ADAM optimiser and showed low computational complexity. The results proved that optimised algorithms could improve diagnostic performance while maintaining manageable computational requirements. The research lacked explainability analysis, which stands as necessary for clinical application, but retained high diagnostic performance through exclusive CNN utilisation, and its feature extraction remained limited by this choice.

The research of Kassim et al. described RBCNet, a dual deep learning architecture which integrates U-Net and Faster R-CNN to identify red blood cells in densely packed microscopy images [15]. Their method achieved over 97% accuracy by resolving dense image features and cell overlapping problems to produce strong segmentation and detection capabilities. The dual architecture demonstrates how complementary systems enhance standard object detection techniques to manage high-density data scenarios.

This work explores the development of a computationally efficient diagnostic system based on ConvNeXt models to address the above-mentioned challenges and provide a scalable, accurate and practical solution to malaria diagnosis. It uses the ConvNeXt architecture to solve data scarcity issues while enhancing computational efficiency for robust malaria diagnostics across resource-poor environments. The ConvNeXt model demonstrates potential as an advanced replacement for existing state-of-the-art models such as ResNet and Swin Transformer through its integrated approach of convolutional efficiency and vision transformer-based hierarchical feature extraction.

The objectives of this study are as follows:

- To build a robust automated malaria diagnostic tool using the ConvNeXt architecture.
- To improve performance using transfer learning from pre-trained models on large datasets such as ImageNet.
- To investigate how data augmentation can make the model more robust in heterogeneous and low-resource situations.
- To evaluate the performance of ConvNeXt compared to other state-of-the-art architectures such as ResNet and Swin Transformer in terms of accuracy and computational efficiency.

**Fig 1:**
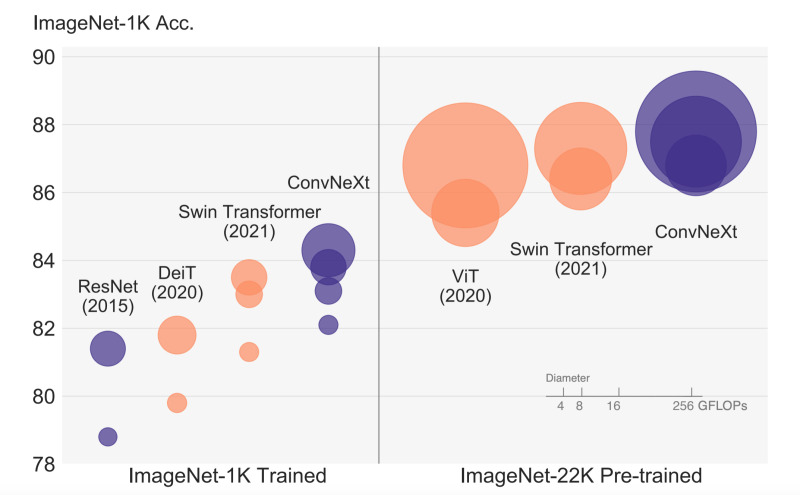
Performance of ConvNext on ImageNet. Source: [16].

In Fig 1, it is observed that the proposed ConvNeXt, which is a modernised convolutional neural network architecture, outperforms other current complex models such as ResNet, DeiT, and Swin Transformer in both accurate training and computational efficiency when trained on ImageNet datasets [16]. The ConvNeXt architecture employs a streamlined convolutional design, using innovations such as larger kernel sizes, Layer Normalisation (LayerNorm), and pre-trained weights from large datasets like ImageNet-22k [17]. These features enable the model to achieve high accuracy with reduced computational resource needs. Compared to ResNet and Swin Transformer, the accuracy of the ConvNeXt is slightly higher, while using fewer FLOPs. This highly optimised system has a specific hierarchical feature extraction function that makes it faster and more accurate than standard CNNs and transformer-based models during the training and testing processes.

Given the scarcity of big medical-labelled datasets, transfer learning and data augmentation have been extensively used in this work. Transfer learning is a technique that enables the models to use the knowledge learned from one task and apply it to another task, such as malaria detection from images. Conversely, the data augmentation technique enhances the dataset’s size by manipulating the image conditions to make the model less sensitive to practical variations.

This study explores a novel application of the ConvNeXt architecture, combining transfer learning with advanced data augmentation techniques for medical imaging. In contrast to conventional deep learning, feature extraction capabilities can be improved by transferring knowledge from the large-scale ImageNet dataset despite the relatively small dataset, used in this study. Moreover, integrating explainable AI tools such as LIME and LLaMA brings a unique dimension to a diagnostic process where the model’s decision-making can be visually and textually interpreted. These innovations highlight the opportunities for AI-driven systems to enhance diagnosis accuracy while promoting greater clinician acceptance by being transparent in AI predictions.

## 2. Literature review

### Current challenges and limitations

Malaria is still a public health problem in many areas of the world, especially in sub‐Saharan Africa, where delayed or incorrect diagnosis aggravates malaria mortality and morbidity rates [1,2,18]. Currently, the primary diagnostic method for the detection of parasitic infections is the manual microscopic examination of blood smears by experienced personnel, which is functional and suitable for use in a controlled laboratory setting; however, this technique has a number of limitations, including its operator dependency, the time it takes for diagnoses, and large result variability that is mostly a result of operator error [19,20]. Consequently, there is increasing interest in minimising the dependency on manual processes and enhancing diagnostic consistency through the development of automated diagnostic systems.

### The role of deep learning in medical image analysis

Advances in medical image analysis using artificial intelligence, particularly deep learning, have provided new opportunities for automating diagnostic processes in healthcare. Convolutional neural networks (CNNs) have been found to be effective for image-based tasks such as image classification and object recognition suitable for medical diagnostics [21,22]. [13,14] have shown that CNNs can accurately classify infected and uninfected red blood cells in thin blood smears.

Kumar’s model achieved an accuracy of 96.62% using CNN-based techniques combined with optimisers such as SGD and Adam. This is strong evidence that CNN-based diagnostic workflow automation is beneficial while maintaining high accuracy. The large amounts of labelled data required by standard CNNs, which are often unavailable in medical contexts, pose a significant challenge for CNNs [23]. As a result, data scarcity and heterogeneity limit the generalisation ability of these models, highlighting the need for alternative approaches such as transfer learning and data augmentation [24].

### Addressing data limitations through transfer learning and data augmentation

The process of transfer learning allows models to use information from datasets such as ImageNet to perform well at smaller domain-centred tasks [24]. According to [13] the effectiveness of transfer learning across various applications does not deny specialised medical image-focused CNN architectures achieving superior performance compared to general transfer learning models.

In addition, data augmentation plays a vital part in enhancing the model’s robustness [24]. This technique involves generating new synthetic data using transformations of the original dataset such as rotation, scaling, and flipping to increase data diversity. In this study, the original dataset of 27,558 images was expanded to 606,276 augmented samples for training, enhancing the ability of the model to generalise well to different imaging conditions and reduce the risk of overfitting.

### Advancements in CNN Architectures: ConvNeXt

ConvNeXt is a modern version of convolutional neural networks (CNNs) combining the progress of both traditional CNNs as well as transformer-based models [17,25]. ConvNeXt was originally intended to revisit the potential of convolutional models in computer vision through a series of key innovations for feature extraction and, at the same time, for computational efficiency [25].

One of these innovations is using larger kernels, which provide global context in the layers to increase their capacity to find complex spatial interrelationships within images [26]. Layer normalisation is another significant improvement which makes training more stable by normalising input data per layer and thus reduces internal covariate shift and increases convergence rate [25]. In addition, the architecture uses large-scale pre-trained weights from ImageNet 22k so that it can use an ample amount of prior knowledge without consuming a substantial amount of task-specific labelled data [27,28].

These improvements position ConvNeXt as a competitive architecture for medical image analysis where labelled data may be scarce and computational efficiency is an important factor to consider. ConvNeXt combines the scalability and simplicity of traditional CNNs with features that augment representational capacity to give an alternative to promising yet resource-intensive vision transformers.

### The Swin Transformer Architecture

Swin Transformer is the first vision transformer that relies on hierarchical self-attention over spatial complexities present in image data [29]. In contrast to classical CNNs, Swin Transformers partition the image into non-overlapping patches, perform multi-head self-attention inside and across the patches in a progressively hierarchical way, and finally learn a tree structure for the representation from the aggregated sub-patches [30,31].

Additionally, it has multi-scale feature representation indispensable for tasks involving complex visual patterns. However, this incurs increased resource demand and slower inference times, especially as inputs are scaled to larger input resolutions. In clinical settings, time-sensitive diagnosis and computation resources limitations are commonplace and might make models with high computational overhead, such as Swin Transformer, difficult to use.

### Explainability of AI-based diagnostics

Deep learning models face their main obstacle for widespread medical adoption because they function as black boxes due to their hidden processes [32,33]. The need for models that provide explanations to support clinical trust, and decision-making is essential for clinicians to accept predictions. This study uses explainable AI (XAI) frameworks, namely Local Interpretable Model-Agnostic Explanations (LIME) and Large Language Model Alignment (LLaMA), to address this challenge [34].

The combination of LIME along with LLaMA enables healthcare professionals to receive visual explanations of model decisions through identified image areas and textual descriptions of the prediction process to transform complex artificial intelligence outputs into healthcare-friendly insights [34–36]. The explainability tools improve transparency and build trust among medical practitioners in AI-driven diagnostic methods, facilitating successful healthcare workflow adoption.

### Optimisation techniques

Obtaining high generalisation in deep learning models to ensure reliable performance in unseen and diverse data is critical [37,38]. Optimisation strategies are needed to find a good balance between model complexity, training efficiency, and robustness to data variation [39]. The application of one such technique, namely, label smoothing, assists in the prevention of the problem of overfitting, in which the model becomes too confident in its predictions and memorises training data rather than generalising it [40]. Label smoothing alters the target probability distribution by lightly decreasing the probability of the label given by the exact one, and a small fraction of probability of the labels occurs [40,41]. This adjustment helps to keep the model just a little uncertain, which makes for less sensitivity of the model to noise, imbalances in the dataset [42]. Label smoothing improves the model’s capacity to generalise to real-world data with imaging condition variation and noise by reducing overconfidence.

AdamW optimiser was also used to increase the training efficiency and stability of the model [43]. An advanced version of Adam optimiser, which decouples the operation of weight decay from gradient update, is called AdamW. Adam and other traditional optimisers may update parameters in a way that causes the magnitudes of the weights to increase excessively, potentially leading to overfitting [43,44]. This is addressed by AdamW, which independently manages weight decay without influencing gradient-based learning by applying a regularisation term. As a result, the parameter updates are more controlled and lead to faster convergence of the model with fewer possible values for the parameters [44,45]. This additionally allows for better hyperparameter tuning, as decoupling weight regularisation from the learning rate reduces the likelihood of training instability.

A combination of these optimisation techniques, label smoothing and AdamW, are essential to the optimality of a model. They enhance its robustness when trained but also retain the capacity to achieve high accuracy and generalisability upon the evaluation of the model on external validation and test datasets [40,45]. These optimisations help the model perform exceptionally well while reducing the risk of overfitting. They strike a balance between maintaining general performance and ensuring reliability, making the model suitable for deployment in clinical environments, where data may vary in quality and consistency.

### Contributions

Deep learning models have resulted in significant progress for automated malaria diagnosis; however, some challenges remain in terms of data scarcity, model robustness and interpretability. Existing studies indicate that the performance of the models is hindered due to limited access to the annotated medical datasets [32,46]. To compensate for this, data augmentation is used to boost diversity and the amount of training samples. For instance, there is an indication that previous studies have expanded the original datasets by image augmentation and found that the newly generated images could boost the models in adapting to varying conditions.

Transfer learning has proven to be useful in improving the model performance with limited medical data, especially using pre trained models from large datasets like ImageNet [47]. Research has observed that deep learning models can use transfer learning to accelerate convergence and increase generalisation across many disparate medical imaging tasks, enabling the models to overcome the problem of limited datasets [48,49]. This approach has been shown to improve diagnostic imaging tasks in studies using convolutional architectures such as ResNet and ConvNeXt in the task of malaria detection.

The issue of the interpretability of models is particularly critical in the context of applying AI in healthcare [50]. However, to aid human understanding of model predictions, explainable AI (XAI) frameworks, including Local Interpretable Model-Agnostic Explanations (LIME), are being used. Some research also points to the fact that the inclusion of XAI tools into models can be the cornerstone for trusting the model and accepting its use among clinicians because it gives an insight into the diagnostic process [50,51]. In particular, the interpretability of this approach is highly important, especially in environments where healthcare professionals need to understand and justify automation to ensure patient safety.

In comparing hierarchical transformer models and convolutional neural networks, some studies indicate contrasting aspects [52,53]. While transformers are effective at capturing complex spatial dependencies through self-attention mechanisms, convolutional models, especially those optimised with larger kernels and pre-trained weights, generally achieve higher computational efficiency. ConvNeXt models have been found to be robust across a range of clinical conditions as they have been shown to be cost-effective and scalable, which makes them more suitable for malaria diagnosis [54–56]. This study builds on these findings by optimising ConvNeXt models for malaria detection in resource-limited settings.

## 3. Materials and methods

Table 1 summarises the methodology used in this study, detailing the key components involved in data preparation, image preprocessing, augmentation, algorithm development, and deployment.

**Table 1:** Summary of the methodology.

Section	Subsection	Details
Dataset Acquisition	Data Source	LHNCBC Collection
	Images	27,558 total (13,779 Parasitised + 13,779 Uninfected)
	Characteristics	Giemsa-stained, variations in lighting, contrast
	Ethical Approvals	NLM IRB & SCiiSREC
Image Preprocessing	Image Resizing	Resized to 224x224 using Lanczos Interpolation
	Image Normalisation	Pixel values centered (Mean = 0, Variance = 1)
Data Augmentation	Techniques	Flipping, rotation, scaling, Gaussian noise, contrast adjustments, etc.
	Result	Dataset expanded to 606,276 images
Algorithms	Swin Transformer	Multi-Head Self-Attention
	ResNet (18 & 50)	Residual Learning
	ConvNeXt	Enhanced CNN with label smoothing, AdamW optimisation
Model Development	Transfer Learning	Pre-trained on ImageNet
	Fine-Tuning	Customised for Malaria Classification
	Training	Mixed Precision, OneCycleLR Scheduler
Model Application	Gradio App	Real-time malaria diagnosis
	Ensemble Models	Combines ConvNeXt Tiny & ConvNeXt V2 Remod
	Explainability	LIME Heatmaps & LLaMA Descriptions
Deployment	Application Design	Portable, resource-efficient system

### Dataset acquisition

The dataset used for training and evaluating the deep learning models in this study was obtained from the Lister Hill National Center for Biomedical Communications (LHNCBC), part of the National Library of Medicine (NLM), which hosts a publicly available collection of malaria-infected blood smear images, available at [57]. This dataset was initially collected at the Chittagong Medical College Hospital in Bangladesh, and the data are made up of thin blood smear images [58]. The dataset comprises 27,558 images evenly distributed between parasitised and uninfected samples, with each category containing 13,779 images.

This balanced distribution ensures the reliability and validity of subsequent analyses and research findings [59]. It prevents the model from being trained to overemphasise one class over the other in cases where the dataset could be more balanced between the two categories.

The blood smear images were photographed using a smartphone camera, which was held up to the eyepiece of a microscope; this configuration mimics the conditions likely to be found in low-resource settings. All the images were obtained from the blood smears stained with giemsa, the standard method of malaria diagnosis by microscopy. The images were then reviewed and labelled by expert technicians to determine whether or not the parasites were present. Each image had a dimension of 5312x2988 pixels, with the circular area depicted as the view through the microscope lens. As a result of the limited resources used in the imaging of blood smears and the variability in the preparation of the samples, the images had variations in lighting, contrast, and colour balance.

To avoid any possibility of identifying patients who may be reflected in the dataset, the patient data were de-identified before online publication. The Institutional Review Board (IRB) approved using the data at the NLM (IRB#12972) [58]. Additionally, this study received ethical clearance from the University of Johannesburg’s School of Consumer Intelligence and Information Systems Research Ethics Committee (SCiiSREC) under ethical clearance code 2024SCiiS040. This clearance is valid for three years, starting on 1 August 2024. This approval ensures that the use of the data complies with the set ethical procedures for handling and analysing medical data.

In Table 2, the image counts are supplemented with both categories’ mean and standard deviation.

**Table 2:** Image statistics for parasitised and uninfected categories.

Category	Image Count	Mean (R, G, B)	Standard Deviation (R, G, B)
Parasitised	13,779	[0.4507, 0.3882, 0.3955]	[0.3109, 0.2954, 0.2656]
Uninfected	13,779	[0.4478, 0.4841, 0.4634]	[0.2966, 0.3222, 0.2919]

The mean and standard deviation values are pixel intensities, which are the extent of brightness or colour of a pixel on an image [60]. The pixel intensity values are from red, green, and blue (RGB). These channels amount to the image’s colour, which ranges from 0, representing black, to 1, representing white. These are colour measurements for the pictures and consist of average colour intensities of the parasitised and uninfected samples regarding brightness and colour changes [61]. As the value in a channel increases, the corresponding pixel intensity value will increase, implying a light and or intense colouration.

Pixel intensity analysis has been applied to detect parasites within microscopic images, particularly in identifying infected and uninfected cells. Studies such as [61] have shown the use of pixel intensities for classifying stained microscopy images based on colour variations. Similarly, several studies have demonstrated that pixel intensities are useful in diagnosing parasitic conditions [62–65]. These works provide solid grounds for using pixel intensities, particularly in automated diagnostic systems.

The average pixel intensity of the colour channels in the “Parasitised” images is Red = 0.4507, Green = 0.3882, Blue = 0.3955. This indicates that the ‘Parasitised’ images have moderate pixel intensity levels in these channels. The standard deviations for the same set of images are approximately 0.3109, 0.2954 and 0.2656, indicating the pixels’ spread of intensity values. A larger standard deviation indicates greater dispersion; in this case, the dispersion is relatively moderate across the colour channels, which means there is some difference in image quality or staining intensity but not too extensive.

The pixel intensities for the “Uninfected” images are 0.4478, 0.4841, and 0.4634 in the three colour channels. These values show slightly higher pixel intensity, especially in the Green and Blue channels, than the “Parasitised” images. The standard deviations for the “Uninfected” images, which are 0.2966, 0.3222, and 0.2919, also indicate that the pixel intensity is moderately dispersed, similar to the “Parasitised” images but with variability across the channels. This dataset is well-balanced and statistically stable, which allows for the practical training of malaria detection models.

### Image pre-processing

#### Image resizing.

The data used in this study were pre-processed before being applied to the deep-learning models used in this study. The input size of the original images at a resolution of 5312x2988 pixels offered an abundance of visual information; however, they were computationally costly and were not appropriate for the input dimensions of most current deep learning architectures. To this end, all images were resized to 224 × 224 pixels as most of the current ConvNeXt, Swin Transformer, and ResNet models have a standard input image size of 224 × 224 pixels [66].

These steps are essential, especially in resizing the images, because this has an impact on the speed with which the models can process the data; it lowers the memory usage during the process and allows the training of the models to be more efficient without losing much detailed information that is important in identifying malaria parasites. This resizing step also aligns with the study’s objective of making the system computationally efficient and suitable for resource-limited settings. However, in most cases, when images are resized, they tend to lose some details, which can be crucial, especially when the resizing is performed to a great extent, such as here [67].

To avoid loss of information during image resizing, the Lanczos interpolation filter was used during the resizing of the image in Python, a method used for image scaling when the quality of the image should be preserved when downscaled [68,69].

Lanczos interpolation is employed to resize the images while preserving the edges and fine details of the images by using the ‘sinc’ function so that the morphology of malaria parasites is well retained in the resized images [68,70]. Thus, applying this method, the details of the pre-processed images were preserved, which is crucial for accurately detecting and classifying the parasitised and uninfected blood smears by the deep learning models. Lanczos interpolation helps resize images while keeping important medical details, solving the problem of image quality loss that can impact classification accuracy. Hence, despite the lower image resolution, which helped to enhance computational speed, the Lanczos filter was used to maintain an adequate image quality for the intended application.

Sinc or sinus cardinalis is a mathematical function applied in signal processing, especially in image processing for interpolation, especially in resampling or resizing images [71]. The sinc function is defined as:


$$sinc\left(x\right)=\frac{\sin\left(\pi x\right)}{\pi x}\;$$ (1)

The sinc function, shown in Equation 1, is a continuous and periodic function defined by oscillations and decreases when moving away from zero [72]. The sinc function is essential in Fourier analysis and can be described as the ideal low-pass filter in the frequency domain. It avoids the problem of aliasing, where different signals appear identical when sampled and produce distorted resampled images or signals.

This study uses the sinc function in Lanczos interpolation, which calculates new pixel colours while an image is being scaled. The sinc function decays and can be used as a weighting function when resampling surrounding pixel values, which produces much smoother transitions between pixels [73]. This allows high-frequency details, such as sharp edges, to be kept when the image size is decreased, which is essential when dealing with the images of blood smears used in malaria detection. The resizing process is aimed at satisfying the pretrained models’ image size requirement while preserving essential features, allowing for high diagnostic accuracy despite computational constraints. This approach ensures that important characteristics are maintained, leading to more reliable and accurate results in the analysis.

#### Image normalisation.

After resizing the images, the normalisation process was performed to ensure that pixel intensities were consistent in the dataset. Normalisation is an essential procedure in deep learning since it enhances the stability of the learning process by preventing variations in the properties of the input data [74,75]. For the normalisation, the mean and the standard deviation of the pixel values of the images in each of the three colour channels, namely the red, the green and the blue, were computed.

The numerical results of the calculated mean values are listed in Table 1, are 0.4507 for the red channel, 0.3882 for the green channel and 0.3955 for the blue channel; the standard deviation values are 0.3109, 0.2954 and 0.2656, respectively. These values were then applied to normalise each image to ensure their pixel values had a mean around zero and the variance was one. The normalisation process improves the stability of the model by ensuring that the data is consistently prepared and standardised. Furthermore, it aligns the pre-processing closely with the objective of developing a reliable diagnostic system which effectively manages and interprets images captured under a range of diverse and challenging conditions, thereby enhancing its overall performance and accuracy. Additionally, this pre-processing step is essential to aid the models in learning and performing well when faced with data they have not encountered before by normalising the brightness and contrast of the images.

### Data augmentation

Data augmentation is significant because it improves the transferability of the deep-learning models for malaria parasite identification [76]. The lack of large, high-quality datasets is a common challenge in low-resource settings. Thus, data augmentation is useful in artificially increasing the amount of available data and introducing variability typical in real-life conditions [77,78]. Data augmentation addresses the challenges posed by limited datasets. This technique enhances the variety of training examples available, allowing models to learn from a broader range of conditions and variations. As a result, the predictions made by these models become more robust and reliable, improving their performance in practical applications. To define the models and make them capable of identifying the malaria parasites in varied situations, many data augmentation operations were performed on the base images, thereby creating a large dataset.

In this study, the techniques used for data augmentation were used for imitating diverse conditions under which blood smear images may be taken. Horizontal flipping applied the transformation that reflected the images across the x-axis, and vertical flipping was the transformation that reflected the images along the y-axis [79]. These steps were critical to enable the model to learn how to identify malaria parasites, irrespective of their orientation. This step is essential considering the frequent inconsistencies in how blood smear slides are placed on microscopes in practical diagnostic environments.

Rotation is another transformation, where images were rotated at an angle of 45 degrees [80,81]. While preparing and analysing blood smears, they may not always be appropriately placed; hence, the model should be able to deal with rotated images. Scaling was used to control the size of the images with a scaling factor of 0.5 to 1.5 [80]. This enhancement was functional in modelling the different magnification levels because the observed visual characteristics may range in size based on the microscope’s settings. Training on scaled images improves the model’s capability to adapt to different magnification levels and aids the models to detect parasites regardless of their size.

Gaussian noise was added to the images to simulate potential noise observable in real-world image acquisition. The noise levels varied from 0.01 to 0.05 times the maximum pixel intensity value, which is 255 [80,82]. This made the training images similar to those taken in conditions that are not favourable, such as low light or inadequate focus of the camera. Contrast adjustments were made, whereby the contrast values were changed by a factor of 0.8 to 1.2 [80]. Using contrast-enhanced images, the model could learn and distinguish parasites even in different image contrasts, thus improving its performance in different diagnostic conditions. These adjustments are essential to accommodate the variations in staining quality and the diverse lighting conditions that are often encountered in diagnostic laboratories. By addressing these factors, we can ensure more accurate and reliable results in the diagnostic process.

Besides these fundamental transformations, some more specific affine transformations were used, such as shearing [80], which changes the image along the x- or the y-axis to mimic some shifting or distortion that may occur while preparing the slides. Several techniques of blurring the images were used to make the images appear out of focus. Gaussian blurring with a sigma range of 0.0 to 3.0 was applied to the images [83]. These techniques imitated the conditions of blurred images due to improper focus on the microscope, and thus, the model learned to recognise parasites in somewhat blurred images. These augmentations enhance the model’s capability to handle challenges posed by suboptimal imaging conditions, which frequently arise in low-resource settings. This ensures that the model remains reliable and accurate when high-quality imaging is not available.

Sharpening was applied to the images to make the details more apparent, with the alpha range from 0 to 1 and lightness range from 0.75 to 1.5 [84]. Sharpening improves the details in the images, especially the edges of the parasites, which are very important in identification. Colour-based augmentations, such as changes to hue and saturation, were also incorporated [85]. These adjustments were informed by differences in staining method when preparing blood smears and differences in lighting or imaging systems. Elastic transformation and dropout were also applied, introducing more variability. The elastic deformations, which were controlled by alpha = 50 and sigma = 5, caused minor shifting of the image, and this was beneficial as the model could identify parasites even if there was a slight deformation in the image [86,87]. Elastic deformations reflect the variations that occur during the manual preparation of slides, highlighting the inconsistencies that can arise from this process.

Cropping was performed randomly by removing parts of the image with 0.02 to 0.1 pixels [88]. This transformation aided in the model being more generalised so that it could still predict the images even if some parts of the images were cut or blurred. Channel shuffling was applied with a probability of 0.35, where the colour channels of the images were randomly interchanged to cope with colour channel variations [89]. These techniques ensure that the model is capable of handling unpredictable changes in imaging configurations, such as altered microscope settings or incomplete slides.

The use of the imgaug library was employed to apply a broad but medically restricted set of augmentations to improve real-world model robustness to microscopy variability. Specifically, these included horizontal/vertical flipping, affine transformation (±45° rotation, scaling 0.5x to 1.5x, shearing), Gaussian and average blur (σ ≤ 3.0), dropout, contrast and brightness variation (1.2x to 0.8x), elastic and perspective deformation and controlled hue/ saturation shift (±20).

The parameter selection for all parameters was made to realistically simulate differences encountered during slide preparation, staining, or imaging. The augmentations that were used maintained essential cellular morphology and minimised distortion, which could compromise diagnostic integrity. This process adheres to standard practices in medical image augmentation [90,91].

Applying these transformations increased the dataset to 606,276 samples, where 303,138 is the number of parasitised samples and 303,138 is the number of uninfected samples. The mean pixel values for the parasitised images were proposed to be [0.44839746, 0.38788548, 0.39583275] while those of the uninfected images were [0.4479274, 0.4817927, 0.46273437]. The standard deviations were 0.30283427, 0.28804937 and 0.26085448 for the parasitised category, while those of the uninfected category were 0.2897945, 0.313396 and 0.285556. These statistics demonstrate the effects of the augmentation process that made the dataset more extensive and diverse in terms of the images’ features.

**Fig 2:**
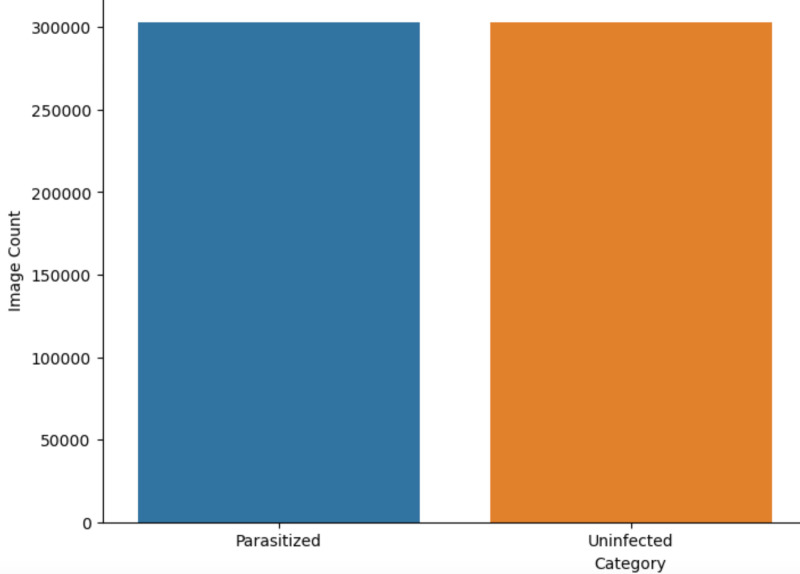
Image count per category (After data augmentation).

Fig 2 visually represents the dataset after data augmentation, explicitly comparing the number of images in two categories: The two main groups used in this study were parasitised and uninfected. There are two categories in total, each of them including about 303,138 images, proving the balanced distribution of the dataset after the augmentation process. This is very important in training the machine learning models, particularly in classification problems, to avoid being influenced by one category due to data sampling.

**Fig 3:**
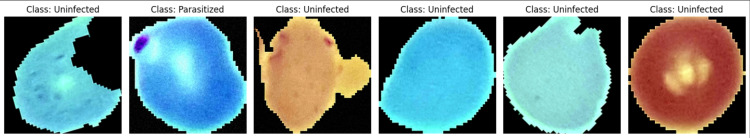
Sample images after data augmentation.

The augmentation made it possible to introduce variability in the form of geometric transformations, noise, scaling and colour variations, among others, as shown in Fig 3. This was performed by adding more training images and mimicking many scenarios one might encounter when capturing blood smears in practice, thus improving the generalisation capability of the models. Such augmentation aims to ensure that the models can identify the malaria parasites identifiable across various images and illumination and rotations, which are common in real-world applications.

Table 3 summarises the basic descriptive statistics of the characteristics of the augmented dataset used to train the malaria detection models. As seen in Table 3, the statistics of the dataset are reasonably even, and pixel intensity and variation are slightly different between Parasitised and Uninfected images, which will help the model during training. These colour distribution patterns are significant as they enable the model to distinguish between the parasitised and uninfected blood smear images, thus enhancing the model’s performance.

**Table 3:** Image statistics for parasitised and uninfected categories (after augmentation).

Category	Image Count	Mean (R, G, B)	Standard Deviation (R, G, B)
Parasitised	303,138	[0.44839746, 0.38788548, 0.39583275]	[0.30283427, 0.28804937, 0.26085448]
Uninfected	303,138	[0.4479274, 0.4817927, 0.46273437]	[0.2897945, 0.313396, 0.285556]

### Algorithms

This paper utilised several sophisticated deep-learning models to identify malaria parasites in microscopic blood smear images. These models cover various architectures that effectively capture local details, e.g., parasite shapes, and global information, e.g., cell distributions, in the images. The models used were the Swin Transformer (Swin Tiny), ResNet18, ResNet50, ConvNeXt Tiny, ConvNeXt V2 Tiny, and a modified version of ConvNeXt V2 called ConvNeXt V2 Remod. Every architecture has been chosen to work with high-resolution medical images and, at the same time, employ transfer learning, allowing the model to take knowledge from previously trained models trained on large datasets like ImageNet.

#### Swin Transformer architecture.

**Fig 4:**
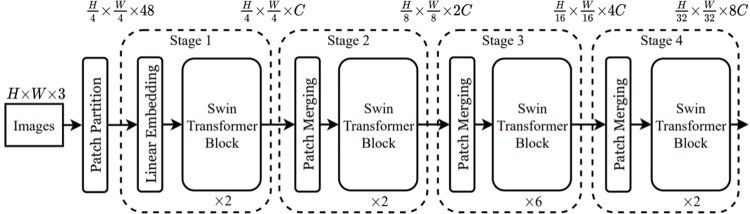
Architecture of a Swin Transformer. Source [29].

This study used the Swin Transformer, particularly the Swin Tiny model, to exploit its window-based multi-head attention [29]. This architecture splits the input images into multiple non-overlapping local windows and then learns the self-attention from these local windows. The Swin Transformer uses this mechanism to analyse information at different scales, which is crucial for spotting subtle patterns in medical images. This way, Swin Transformers can capture regional and global information in images, which is beneficial for tasks such as medical image analysis [92]. The Swin Transformer architecture, as shown in Fig 4, is where images are divided into a sequence of non-overlapping patches. Then, a linear embedding layer is applied to generate patch tokens. The hierarchical design of vision transformers requires a patch merging layer, which operates as a core element within systems like the Swin Transformer [29]. The layer functions to decrease spatial dimensions of the input feature maps while raising the channel depth, which lets the model efficiently obtain detailed local information alongside a comprehensive global context. Traditional vision transformers keep the same image resolution throughout their architecture, which leads to high computational demands, while Swin Transformer provides superior benefits by changing resolutions [29]. Swin Transformer utilises merging operations for multi-scale feature representation to better process different object dimensions in visual data.

The Swin Transformer block functions as an essential building element in the Swin Transformer framework that improves computational efficiency while managing to capture image details across both local and global scales [30]. Traditional vision transformers summon self-attention mechanisms for whole images but the Swin Transformer block slices images into non-overlapping windows to apply self-attention only within these divided segments. The architecture maintains robust feature extraction performance but achieves this at a much lower computational expense. Each Swin Transformer block consists of two main stages: Window-based Multi-Head Self-Attention (W-MSA) and Shifted Window Multi-Head Self-Attention (SW-MSA). The W-MSA mechanism employs self-attention inside fixed-size windows to seize local patterns in an efficient way without increasing computational complexity [30]. The use of self-attention inside fixed windows leads to non-contiguous features across the representation space. The SW-MSA mechanism moves the window partitions minimally between layers to allow information flow between windows, which improves feature continuity in multiple spatial regions.

Additionally, the Swin Transformer has a hierarchical framework which ensures that fine-grained details, such as parasite morphology, and broader structural patterns, such as cell distributions, are equally well-represented [92].

Mathematically, the self-attention mechanism within a Swin Transformer can be represented as:


$$Attention\left(Q,K,V\right)=softmax\left(\frac{QK^{T}}{\sqrt{d_{k}}}\right)V$$ (2)

where Equation 2 represents the queries, keys, and values, respectively, and $d_{k}$ is the dimension of the keys. The model incorporates a relative position bias term that enhances its ability to encode the spatial structure within each window.

Q (queries) and K (keys) and V (values) are distinct linear transform operations on input features which play different parts in the self-attention process. The similarity scores between Q and K elements determine the proportional attention values distributed across image spatial locations. The evaluation process receives scaled similarity scores as input through a softmax function to produce attention weights. A weighted calculation of V computes the values (V) through these weights, thus allowing the model to collect data from various spatial locations across the image.

The Swin Transformer uses this mechanism within this study as it enables precise detection of malaria parasites in blood smear images through its ability to see long-range dependencies and subtle image patterns over distant regions. The model needs to detect slight yet spatially separate features among infected and uninfected red blood cells, as these cells sometimes share visual traits. Through the Q, K and V functions, the attention mechanism allows the model to dynamically select important diagnostic regions, which enhances its ability to distinguish parasitised cells from uninfected cells in visually complex input data. The model achieves better robustness and classification accuracy in malaria diagnosis due to this method.

Swin Tiny was pre-trained on ImageNet-1K, a dataset containing over a million labelled images. It achieved a top-1 accuracy of 81.2% and a top-5 accuracy of 95.5% on ImageNet, with 28 million parameters and a computation cost of 4.5 GFLOPs [93].

The Swin Transformer model provides superior computational efficiency along with competitive accuracy when benchmarked against standard CNN architectures and vision transformer models in image processing applications [94]. Self-attention-based non-overlapping windows enable Swin Transformer to dynamically adapt to hierarchical features, whereas fixed receptive fields remain a limitation in standard CNNs like ResNet. The architecture enables better capture of both local and global dependencies, which improves feature extraction capabilities, leading to advanced accuracy [29,30]. The Swin Transformer shows greater computational demands than lightweight CNNs, including ResNet18, because it uses a transformer-based architecture. Despite their longer training times, the generalisation power and robustness to image quality variations of Swin Transformer models enable high diagnostic precision applications [95]. Depending on the deployment context, Swin Transformer models deliver excellent diagnostic precision yet demand extra optimisation for resource-limited, real-time use cases.

#### ResNet architectures.

The ResNet18 and ResNet50 were selected as the baseline models to be compared with the proposed models. These models are a part of the Residual Networks, which are the types of neural networks developed to address the vanishing gradient problem that is a big challenge in training deep neural networks [96]. Residual learning enhances performance by enabling deeper neural networks to learn effectively while mitigating the degradation problem frequently encountered in complex architectures. The residual block is central to ResNet’s architecture and is expressed mathematically as:


$$y=F\left(x,\left(W_{i}\right)\right)+x$$ (3)

where x is the input, F(x,(W_i_)) is the learned transformation with weights W_i_ and y is the block output, as shown in Equation 3. This structure helps the network to learn identity mapping when deeper layers do not help enhance performance and allow a flow of gradients across many layers.

ResNet18 is a less complex model with 18 layers, while ResNet50 is a deeper model with 50 layers; hence, it can learn more complex data features [97]. ResNet18 was pre-trained on ImageNet and fine-tuned for malaria detection, and the second model was pre-trained on ImageNet and fine-tuned for malaria detection [98]. Although ResNet18 was used as the baseline model, ResNet50 had a deeper network and could thus identify more intricate visual patterns in the blood smear images and differences between them [99]. The use of both shallow architecture, represented by ResNet18, and deep architecture, exemplified by ResNet50, facilitated a comprehensive comparative analysis. This approach highlighted the performance trade-offs, shedding light on the balance between computational efficiency, where less processing power is required, and the capabilities for feature extraction, which refers to the ability to identify and capture intricate patterns in data. This comparison allowed for a deeper understanding of how different model depths impact both speed and accuracy in processing complex datasets.

#### ConvNeXt and ConvNeXt V2 architectures.

ConvNeXt Tiny, a novel architecture based on conventional convolutional neural networks (CNNs), was another critical architecture used in this study [28,100]. ConvNeXt is an advanced model that combines the strengths of convolutional neural networks with the versatility of Transformer architectures [17,25,26]. This integration makes it particularly effective for medical image analysis, where accuracy and adaptability are critical. It uses the spatial hierarchies of convolutional layers and the attention mechanisms of Transformers, to capture intricate patterns in medical imaging data, thereby enhancing diagnostic capabilities and improving patient outcomes. Given its basis upon the framework of Vision Transformers (ViTs), this innovative architecture utilises a hierarchical design that integrates high-level features, such as overall shapes and object relationships, and low-level features, including textures and colours. This combination enhances the model’s ability to process and analyse images with greater depth and accuracy, making it particularly effective for a wide range of imaging tasks and applications.

**Fig 5:**
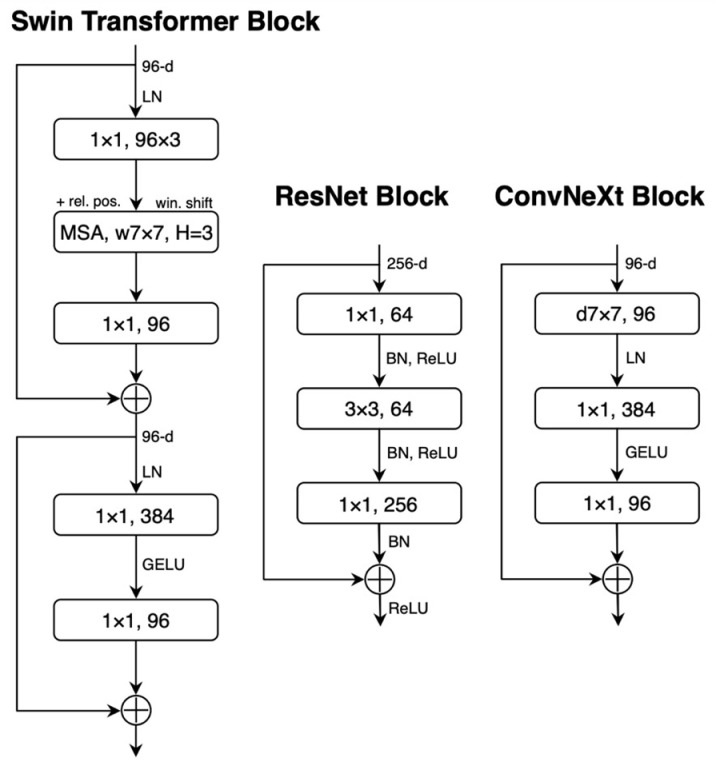
Block designs for the used models. Source [16].

Consequently, the feature extraction of Swin Transformer, ResNet, and ConvNeXt model block designs are different, as shown in Fig 5. Swin Transformer block captures local and global features with multi-head self-attention (MSA) with shifted windows (w7x7), followed by layer normalisation and Gaussian Error Linear Unit (GELU) activation. A ResNet block is designed based on the residual structure of 1x1 and 3x3 convolutions, batch normalisation and Rectified Linear Unit (ReLU) activation to help feature learning. ResNet is modernised to ConvNeXt block by replacing 3x3 convolutions with depthwise convolutions (d7x7) with an extra parameter of channel multiplier, as well as using layer normalisation and GELU activation for better efficiency.

The core operation in ConvNeXt is convolution, mathematically described as:


$$\left(S*K\right)\left(i,j\right)=\sum_{m}\sum_{n}S(i+m,j+n)K\left(m,n\right)$$ (4)

In Equation 4, S represents the input matrix (image), K, in Equation 4, is the convolutional kernel, and (i,j) denotes spatial positions in the image. ConvNeXt Tiny model was pre-trained on ImageNet, with the top-1 accuracy of 82.1% and has 28M parameters and 4.5 GFLOPs of computation. The pre-trained model was downloaded from GitHub and then fine-tuned to the malaria dataset to teach the model the features of detecting malaria parasites.

Following the success of ConvNeXt, ConvNeXt V2 had additional architectural enhancements, including learning rate scheduling and modified activation functions for improving the image classification task performance [100]. These updates refined the model’s capacity to generalise across diverse imaging conditions critical for malaria diagnosis in resource-limited settings. The ConvNeXt V2 Tiny model employed in this study, which was also pre-trained on ImageNet, provided a top-1 accuracy of 83.0 per cent and had a computational complexity of 4.47 GFLOPs with 28. 6 million parameters. Similar to ConvNeXt V2, the model was fine-tuned on the malaria dataset to change the pre-trained weights of the model to adapt to the characteristics of the images of parasitised and uninfected blood smears.

#### ConvNeXt V2 Remod.

This study’s ConvNeXt V2 Remod model was based on the ConvNeXt V2 Tiny architecture. The Remod version introduced tailored enhancements designed to improve performance and accuracy. One notable enhancement is label smoothing, which helps to minimise overfitting, an issue that can arise when a model becomes too focused on the training data. Through the application of label smoothing, the model is better equipped to generalise across unseen data, ultimately leading to improved predictive accuracy. In the training process, label smoothing was used with α = 0.1 [41,42]. Label smoothing shifts the target labels to prevent the model from attaining extreme confidence and helps deter the overfitting of the model [42]. Mathematically, label smoothing modifies the loss function as follows:


$$L_{CE}=-\sum_{i=1}^{N}\left(\left(1-\alpha \right)y_{i}+\frac{\alpha }{C}\right)\log\left({\hat{y}}_{i}\right)$$ (5)

where C is the number of classes (in this case, C = 2 for binary classification), alpha distributes a small part of the probability mass to the incorrect classes, as shown in Equation 5. This study uses an alpha value of 0.1 as the label smoothing factor across the entire set of experiments [42]. The experimental value of the label smoothing factor, alpha, aligns with previous image classification work as it enhances generalisation by making targets softer and reducing overconfidence in predictions [41]. The chosen value maintains class separation while stopping the model from overfitting to particular label targets, ensuring better performance in medical image tasks [101].

**Fig 6:**
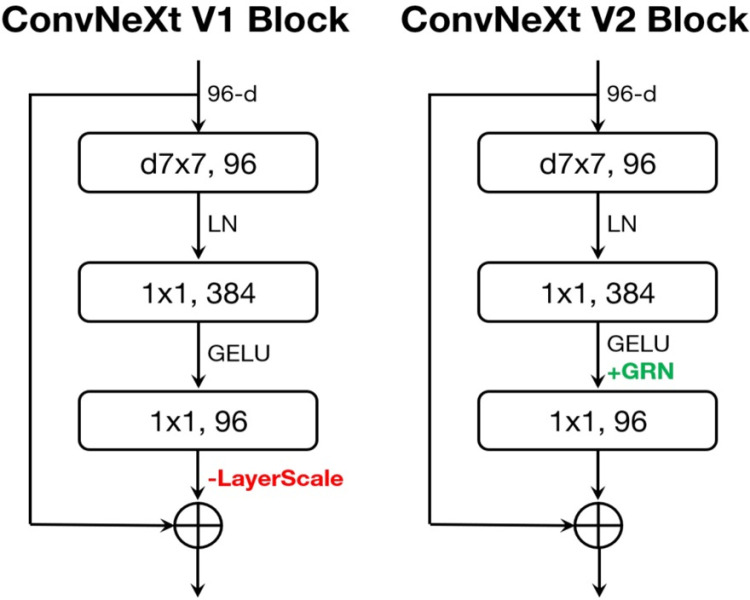
ConvNeXt block designs. Source [100].

The block structures of ConvNeXt V1 and V2, shown in Fig 6, show the core improvements made from one version to the other. The ConvNeXt V1 block consists of depthwise convolutions (d7x7) and then layer normalisation (LN) and GELU activation for efficient feature extraction. The first version of the model introduced LayerScale, which helped stabilise the process. On the other hand, the ConvNeXt V2 block keeps the heart of V1 but introduces Generalised ReLU Normalisation (GRN). This new state-of-the-art normalisation technique enhances the model’s stability and efficacy. This addition of GRN with the elimination of LayerScale boosts the generalisation ability of ConvNeXt V2 across several tasks.

The model’s performance was further improved with the use of advanced optimisation techniques. The AdamW optimiser, shown in Equation 6, was selected for this task as it is a version of the Adam optimiser with weight decay for better generalisation and reduced overfitting [43,102]. AdamW updates the model’s weights using first- and second-order moments of the gradients. The main distinction between Adam and AdamW is that in AdamW, the weight decay is not applied directly to the gradients, which allows the magnitude of the weights to be preserved.

The learning rate was set to 0.0005, and a weight decay of 0.01 was applied to penalise large weights, thus avoiding overfitting the training data [45]. The AdamW optimiser is defined as:


$$m_{t}=\beta_{1}m_{t-1}+\left(1-\beta_{1}\right)g_{t}$$

$$v_{t}=\beta_{2}v_{t-1}+\left(1-\beta_{2}\right)g_{t}^{2}$$

$$\hat{m}t=\frac{m_{t}}{1-\beta_{1}^{t}},\hat{v}t=\frac{v_{t}}{1-\beta_{2}^{t}}$$

$$\theta_{t}=\theta_{t-1}-\alpha \frac{{\hat{m}}_{t}}{\sqrt{{\hat{v}}_{t}}+\epsilon }$$ (6)

where:

g_t_ is the gradient of the loss with respect to the parameters at step t

m_t_ and v_t_ are the moving averages of the gradient and its square, respectively,

theta represents the model parameters at step t

alpha is the learning rate, and

Beta 1, Beta 2 and epsilon are hyperparameters of the optimiser.

To improve learning efficiency, the OneCycleLR scheduler was applied [103]. This scheduler modifies the learning rate throughout training to balance exploration (high learning rate) and refinement (low learning rate). The learning rate is first set to maximum value and then decreases over time, which helps the model avoid local minimum in the initial training phase while fine-tuning the weights when training continues.

This is set to zero initially, rises to a maximum of 0.0005 and then decreases using a cosine annealing schedule, shown in Equation 8, over ten epochs. The cosine annealing formula is given as Equation 7 by:


$$\eta_{t}=\eta_{min}+0.5\left(\eta_{max}-\eta_{min}\right)\left(1+\cos\left(\frac{t}{T}\pi \right)\right)$$ (7)

where:

Eta t is the learning rate at time step t,

Eta max and Eta min are the minimum and maximum learning rates, and

T is the schedule’s total number of time steps (epochs).

To optimise the training of the NVIDIA^®^ Tesla^®^ P100 graphics processing unit (GPU) used in Kaggle, PyTorch’s automatic mixed precision (AMP) was implemented to train in mixed precision. It is a technique where half of the computations are performed in a 16-bit floating-point format, whereas the other half are performed in a 32-bit floating-point format. The gradients are calculated in the FP16 format for the sake of optimisation, while the master weights are stored in the FP32 format for the sake of numerical accuracy. The integration of automatic mixed precision (AMP) into the training process brought improvements in efficiency and speed. This method not only accelerates the training time but also ensures that the numerical accuracy of the model remains intact. As a result, the model can seamlessly handle large-scale and computationally intensive tasks, such as training deep learning networks with vast datasets. Through the optimisation of resource usage and reduction of memory consumption, AMP allows faster convergence.

For this, gradient scaling was used to deal with the differences between FP16 and FP32. Gradient scaling is the process of scaling the gradients by a specific factor to prevent the gradients from being too small (and thus causing underflow) or too large (which will cause overflow). This scaling assists in controlling the training process, especially for deep networks with numerous parameters to be estimated.

The training of the ConvNeXt V2 Remod model was performed for ten epochs, with the learning rate being adjusted in a way that aims at deriving the highest performance. The model was trained with high performance and reliability using mixed precision through automatic mixed precision and with the help of checkpointing. Thus, the optimisation strategies and the advanced architectural features helped to enhance the recognition of malaria parasites in blood smear images at the end of the training process.

### Model development

#### Transfer learning.

This work applied transfer learning, where pre-trained models were obtained from GitHub repositories. Transfer learning offered several advantages as it significantly cut down training time. As the models had previously been trained on large datasets such as ImageNet, the computational power and time needed for fine-tuning the models for malaria classification were considerably lower than that required for training new models. This efficiency is particularly critical in resource-limited settings where computational resources and large labelled datasets are scarce. Secondly, transfer learning played a role in reducing overfitting, which is a big problem when dealing with limited samples. Through pre-training, the models with a large set of images continued to perform well on the malaria classification task due to their generalisation ability.

In addition, transfer learning was used to ensure the models’ accuracy and robustness were not compromised by the new task of distinguishing between parasitised and uninfected blood smears since the models could still rely on their prior experience with identifying general objects in images [47]. This approach was instrumental in resource-limited settings where access to vast amounts of labelled data and powerful computing resources is limited.

Swin Transformers, ResNet 18, ResNet 50, ConvNeXt Tiny and ConvNeXt V2 Tiny were the models employed in this study, all pre-trained on large datasets of generic images. These architectures were carefully chosen due to their demonstrated effectiveness in extracting meaningful features from complex data [25,66,97,100]. Their flexibility allows them to adapt to a wide range of tasks, making them particularly suitable for applications such as medical image classification, where accuracy and precision are crucial. There are several benefits of using these pre-trained models; first, they are already equipped with feature extraction capabilities that can be fine-tuned for a specific task, for instance, malaria parasite detection in microscopic blood smear images [104–106].

The pre-trained Swin Transformer models were acquired from the repository managed by Microsoft and are accessible at [107]. Swin Transformers are well-suited for handling high-resolution images due to their unique mechanism of dividing images into non-overlapping windows, enabling efficient computation of self-attention within each window [29,108]. This approach makes it possible for the model to learn both the image’s local and global features, which is very important during the classification, especially in tasks such as parasite identification.

ConvNeXt and ConvNeXt V2 models used in this work were obtained from the public repositories established by Facebook AI Research. ConvNeXt models can be accessed at [25]. They represent advancements over regular convolutional neural networks (CNNs) with features derived from Vision Transformer architectures [16,28]. ConvNeXt V2, accessible at [27], incorporates improvements that include better resource management and improved feature extraction and, hence, is even more suitable for tasks such as malaria classification [100].

These models were first pre-trained on ImageNet, one of the most commonly used datasets in the computer vision field [48]. The ImageNet dataset is one of the most extensive, with over 14 million labelled images spanning 1,000 categories of objects, including animals, buildings, nature, and food, amongst others [109]. This large amount of data helps these models to learn the features well. Hence, they are ideal for transfer learning applications, such as detecting malaria parasites with limited datasets.

Fine-tuning these models for classifying parasitised and uninfected blood smears involved adjusting the final fully connected layers to output two classifications: The blood smears were either parasitised or uninfected [110]. This customisation ensures the models are optimised for binary classification tasks, improving their accuracy in diagnosing malaria. It is essential to replace the pre-trained models’ last layers with new layers specifically designed for malaria parasite detection. While the pre-trained models are well-trained in the general characteristics of the images, such as edges, patterns, and textures, the final layers can be focused on learning the unique characteristics of the blood smears relevant to malaria diagnosis. In this case, the final layers were replaced with custom layers designed for binary classification, parasitised versus uninfected.

#### Training procedure.

The training process for this study was performed on Kaggle, which offered the use of NVIDIA^®^ Tesla^®^ P100 GPU. This is mainly because of the GPU’s architecture and computational ability for deep learning. The Tesla^®^ P100 is based on NVIDIA^®^’s Pascal architecture, includes 16GB of HBM2 memory and performs 10.6 TeraFLOPS in single-precision floating-point operations per second. Such a computational capability helped handle large image datasets used in this study without experiencing low speeds. The P100 had enough memory to operate on large and high-resolution images so that batch processing of models could be performed faster during training.

The training was performed using mixed-precision training with the help of PyTorch’s automatic mixed precision (AMP) [111]. AMP is a technique which enables deep learning models to train faster and with less memory than standard training [111,112]. AMP works on some parts of the network. For instance, the convolutions are computed using 16-bit floats, while the rest of the model’s weights and gradients are calculated using 32-bit floats to balance between speed and accuracy.

This technique also enhances the speed of training and optimisation of the GPU, especially the Tesla^®^ P100, which performs operations on large datasets with great accuracy. Furthermore, the vanishing and exploding gradient issues that are evident when training deep networks with vast parameter spaces like ConvNeXt or Swin Transformer models are also solved by AMP.

#### Optimisation strategy.

This study opted to use the Adam optimiser, as shown in Table 4, which has been known to be efficient in adjusting the learning rate during training [113]. Adam uses the benefits of AdaGrad and RMSProp optimisations involving gradient averages and second-order moments to adjust the model weights [43,114]. This is advantageous for Adam for handling the sparse gradients and noisy data, which are familiar with medical image data. The learning rate of the optimiser was set to 0.0001 for most models, including ConvNeXt Tiny, ResNet18, and ResNet50, due to its ability to fine-tune deep learning features, especially for medical imaging data that typically contain noise and sparse gradients [115]. A low learning rate is essential to guarantee that the model gives only small weight adjustments to make the best estimate without overfitting highly sensitive models on the loss surface [116]. For the ConvNeXt V2 Remod model, a higher learning rate of 0.0005 was used alongside weight decay of 0.01 to improve model generalisation [117].

**Table 4:** Hyperparameters used for fine-tuning different models.

Model	Learning Rate	Batch Size	Optimiser	Weight Decay	Loss Function	Scheduler	Epochs	Mixed Precision
ConvNeXt Tiny	0.0001	128	AdamW	Not Used	Cross Entropy Loss	None	10	GradScaler & autocast
Swin Transformer	0.0001	128	Adam	Not Used	Cross Entropy Loss	StepLR	10	GradScaler & autocast
ResNet18	0.0001	32	Adam	Not Used	Cross Entropy Loss	None	10	GradScaler & autocast
ResNet50	0.0001	128	Adam	Not Used	Cross Entropy Loss	None	10	GradScaler & autocast
ConvNeXt V2 Tiny	0.0001	128	AdamW	Not Used	Cross Entropy Loss	None	10	GradScaler & autocast
ConvNeXt V2 Remod	0.0005	128	AdamW	0.01	Cross Entropy Loss (Label Smoothing = 0.1)	OneCycleLR (Cosine)	10	GradScaler & autocast

Table 4 reveals that the training was limited to 10 epochs per model since computational resources, on the only freely available platform with GPU resources, Kaggle, where only 30 hours of time on GPU are given weekly, was limited. Due to these limitations, more resource-efficient models were selected; consequently, these are ConvNeXt Tiny instead of ConvNeXt Large, Swin Tiny instead of Swin Large, and ConvNeXt V2 Tiny instead of ConvNeXt V2 Extra Large. The large models are pre-trained on larger ImageNet datasets, making them more robust, although they require more computing resources compared to the tiny models.[25,29,100].

The adaptive learning rate of Adam makes the model converge faster than the normal stochastic gradient descent (SGD) [44]. In this study, it was beneficial for large and elaborate models such as ConvNeXt and Swin Transformer with many parameters. Adam allowed the model to automatically and adaptively set the learning rate for each parameter, which made the model optimise itself and increase the training process’s convergence rate.

#### Gradient scaling and stability.

Gradient scaling was used during the optimisation to avoid training process instabilities [118]. It is a method applied in mixed-precision training to prevent the gradients from becoming too small (vanishing gradients) or too large (exploding gradients) [119]. In large networks, such as those used in this study, the parameter space is also significant, which can result in oscillations during training. Applying gradient scaling before backpropagation helped avoid the potential numerical precision problems common in deep learning models.

A learning rate scheduler was used to stabilise the training process during implementation. A learning rate scheduler adjusts the learning rate during training according to the given performance of a model [120,121]. In this case, the scheduler had a learning rate reduction of 0.1 every seven epochs if the validation loss did not decrease. This technique is very helpful in preventing the model from becoming trapped in the local minima. When the validation loss stalls, reducing the learning rate helps the optimiser fine-tune the model weights and allows the model to perform better and converge. For ConvNeXt V2 Remod, a OneCycleLR (cosine) scheduler was applied to balance fast convergence with the risk of overfitting

#### Loss function: Cross-entropy loss.

The loss function used for this study was the cross-entropy loss function, which is most suitable for the binary classification of the malaria parasite cases (parasitised and uninfected red blood cells) [122]. Cross-entropy loss measures how far the predicted class probabilities are from the actual class labels [123]. It is more useful in classification as it penalises the predicted output that is not in line with the actual output. In the case of ConvNeXt V2 Remod, label smoothing was also applied to the cross-entropy loss to mitigate the effect of noisy labels and improve generalisation. In deep learning classification jobs, label smoothing is a regularising technique, preventing model overfitting and enhancing generalisation capability [41]. The label smoothing approach reallocates portions of the probability mass dedicated to the proper class across every class classification. The application of label smoothing reduces model prediction certainty while enhancing calibration performance [42].

Mathematically, label smoothing is given as:


$$q_{i}^{\prime }=(1-\epsilon )q_{i}+\frac{\epsilon }{C}$$ (8)

where:

q_i_ is the original one-hot encoded label,

q^’^_i_ is the smoothed label,

epsilon is the smoothing factor, and

C is the number of classes.

The formula in Equation 8 blends original one-hot labels with a uniform distribution across all classes while minimising extreme prediction confidence. This study employed the same epsilon value of 0.1 that matched the alpha setting of 0.1 from Equation (5). The single chosen epsilon value enables regularisation to follow the same approach in label generation and loss calculation steps. Previous studies applied epsilon = 0.1 for label smoothing as it enhanced model generalisation and reduced prediction overconfidence, especially in critical domains such as medical image diagnosis [41,42,101].

In this work, the model was trained to predict the likelihood of a given blood smear image being parasitaemic. The output from the final layer in the model is a vector of predicted probabilities, which are compared to the actual class labels, and the weights of the model are updated to minimise this loss function known as cross-entropy loss.

Mathematically, cross-entropy loss is computed as:


$$Loss=-\sum {\mathrm{\backslash nolimits}}_{i=1}^{N}[y_{i}.\log\left(p_{i}\right)+\left(1-y_{i}\right).log\left(1-p_{i}\right)]$$ (9)

where:

N is the number of samples,

y_i_ is the true label (either 0 for uninfected or 1 for parasitised),

p_i_ is the predicted probability for the corresponding class.

In this context, cross-entropy loss, displayed in Equation 9, helps the model to give a high probability to the correct class and a low probability to the incorrect class [124]. Since the dataset has equal parasitised and uninfected images, cross-entropy loss prevents the model from leaning towards one class and produces equally good results.

AdamW represents an enhanced optimisation method built on standard Adam which splits weight decay operations from gradient-based parameter modifications [45]. The conventional Adam algorithm uses momentum alongside adaptive learning rates yet applies L2 regularisation to gradients, producing possible suboptimal weight adjustments [14]. AdamW implements distinct weight decay functionality from gradient updates thus preventing the interference of adaptive moment estimates during regularisation. The model separation technique stops model fitting from exceeding its generalisation abilities particularly when dealing with deep learning techniques. The model deployed AdamW during this investigation which delivered stable convergence alongside controlled weight magnitudes and generalised performance across different image conditions. The optimisation approach matches the research goal to enhance malaria diagnosis precision without sacrificing computational speed.

#### Computational feasibility.

Given the typical resource constraints of malaria-endemic regions, the consideration of computational feasibility of deploying deep learning models in these areas is a critical factor. In this study, compact architectures were selected, namely ConvNeXt Tiny and ConvNeXt V2 Tiny Remod, as they have competitive accuracy with relatively lower computational overheads compared to their larger counterparts, such as Swin Large or ConvNeXt XL. Consequently, these models can be trained and run on mid-range GPUs, such as the one used in this study, namely NVIDIA Tesla P100, available through Kaggle, with a memory requirement of less than 30 million parameters and less than 4.5 GFLOPs memory requirement.

This study further employed mixed precision training with PyTorch’s automatic mixed precision (AMP), which allows combining 16-bit and 32-bit floating point operations to further reduce resource usage and speed up training. Highlighting the practicality of the approach under both time and hardware limitations, the training process was completed within Kaggle’s 30-hour weekly GPU quota. The resource-aware model design and the optimisation strategy establish the proposed diagnostic framework as deployable in limited-resource healthcare settings.

### Model application and deployment

To enhance the applicability of the trained ConvNeXt models, a web application was developed using Gradio to facilitate the real-time identification of malaria parasites in blood smear images. Gradio, a Python library, was chosen for its simplicity and ability to provide an intuitive interface suitable for resource-constrained environments. The application has an interface where users can upload blood smear images and receive real-time diagnoses in a limited resource environment. It is accessible at [125].

The application (app) was designed with two main functionalities: The proposed solution includes image classification using ensemble models and the explanation of the predictions using the LIME model (Local Interpretable Model-agnostic Explanations) and Llama 3.1 by Facebook Research [126–129]. These functionalities aim to balance diagnostic accuracy with transparency, ensuring that the app is both effective and interpretable.

The application presents novel contributions through the use of an ensemble of deep learning models (ConvNeXt V1 Tiny and ConvNeXt V2 Tiny) that help improve diagnosis performance and reliability. To do this, the app takes the average of these models’ predictions by assigning weights to each of the models to make a more accurate determination on whether a blood smear is parasitised or not. This ensemble approach outperforms the conventional single-model systems employed in medical diagnostics to provide a more accurate result, especially in the limited resource environment [130]. Furthermore, the app employs mixed-precision training with the help of GradScaler, which ensures the high performance with the minimal consumption of resources, which will be beneficial for the areas with poor infrastructure.

One of the main aspects of the app is the explainability element, using LIME for visualisation and LLaMA for textual elaboration [129,131]. LIME gives out visual maps that demonstrate the parts of the image which are used in making the decision, which helps in improving the explainability of the predictions to medical practitioners [131]. LLaMA enables an additional step in explaining the results as it generates human-readable descriptions of the context and, therefore, helps interpret the machine’s predictions [129]. These explainability features together with efficiency in the use of resources make the Malaria Diagnosis App not only unique but also very useful for use in malaria endemic areas.

#### Use of best performing models.

The application combines the strengths of two fine-tuned models, namely ConvNeXt Tiny and ConvNeXt V2 Tiny Remod, through the ensemble method [132]. Ensemble learning enhances the app’s reliability by using the complementary strengths of both models, ConvNeXt Tiny for computational efficiency and ConvNeXt V2 Remod for higher accuracy. This approach combines the prediction of the first model with the second model’s prediction to make the final decision, increasing the overall reliability of the decision made [133]. From the two models used in this study, ConvNeXt Tiny was purposely selected for its computation efficiency, which allows it to process images at high speed. At the same time, ConvNeXt V2 Tiny Remod was chosen because of its higher accuracy and precision, as highlighted in the comparative analysis.

Every image the user uploads is first processed through a pre-processing step, which includes resizing the image to 224 x 224 pixels and normalising the image using the mean and standard deviation of the malaria dataset. This check helps meet the conditions the trained models expect as input data. The image is then passed through both models, and the average output of the two models is computed. The final decision on whether a blood smear is parasitised or uninfected is based on the average of the two models presented in this work. This approach enhances the diagnostic performance and decreases the rates of false-positive results, thus making the diagnosis more accurate.

#### Model explanation with LIME.

To further enhance the transparency and interpretability of the malaria parasite detection models, the LIME (Local Interpretable Model-agnostic Explanations) algorithm was employed. LIME works by perturbing the input data – in this case, the blood smear images -and observing how the model’s predictions change in response to these perturbations. The algorithm generates explanations by locally approximating the model’s decision boundary and identifying the regions within the image that most influence the final classification. This process is precious for understanding deep learning models, often called “black boxes.”

Mathematically, LIME can be understood as follows:

Using a kernel function, LIME approximates the complex model around the local region of the explained instance: $\pi \left(x_{0},\;x^{\prime }\right)$.

LIME minimises the following loss function to generate explanations:


$$\xi \left(x\right)=\underset{g\in G}{argmin}L\left(f,g,\;\pi_{x}\right)+\Omega \left(g\right)\;$$ (10)

where:

Xi(x) is the explanation for the instance x,

G is the class of interpretable models, L(f,g,Pi_x_), in Equation 10, is the loss function that measures the fidelity of the interpretable model g to the complex model f in the local region defined by the kernel Pi_x_.

Omega(g) is a complexity term that penalises the complexity of the interpretable model g, ensuring that the explanation remains simple and human-readable.

The kernel function Pi(x_0_,x’) assigns higher weights to perturbed samples closer to the original instance x_0_ ensuring that the explanation focuses on the local behaviour of the model. In the case of image classification, this kernel function is often defined based on the Euclidean distance between the perturbed image $x^{\prime }$ and the original image x_0_.

Once the simpler model g(x) is trained to highlight the regions in the image most responsible for the model’s prediction. These highlighted regions correspond to the image features—such as ring-shaped structures or abnormal cell morphologies that the model associates with malaria parasites.

For each blood smear image analysed, LIME produces a visual heatmap highlighting the most influential regions in the classification decision. These highlighted areas allow healthcare professionals to understand why the model classified an image as parasitised or uninfected. This feature is crucial in clinical settings because it provides clinicians with tangible visual cues to validate the model’s predictions. It bridges the gap between complex machine learning models and human interpretability, making AI-driven diagnostic systems more transparent and trustworthy.

By offering healthcare professionals clear, visual evidence of what the model “sees,” LIME enhances decision-making support. Physicians and laboratory technicians are given the output of the model and the rationale behind the decision, building trust in the diagnostic process. Moreover, the mathematical framework behind LIME ensures that the explanations are robust and focused on the most significant aspects of the model’s behaviour.

#### Integration with LLaMA for diagnostic insights.

The above outputs were complemented with textual descriptions produced by the Large Language Model Meta AI (LLaMA) language model to provide more specific case information [129]. It enabled the app to classify blood smear images and briefly describe each classification based on context. An advantage of the system was that it used a pre-trained LLaMA model to provide interpretations in simple language of why a particular classification of the image as parasitised or uninfected was made.

In the case of blood smears classified as parasitised, the LLaMA model offered some understanding of the visual cues that contributed to this classification. It also showed the presence of trophozoites, the asexual form of malaria parasite presenting as ring-shaped structures within red blood cells and some other abnormalities, including irregular shapes of the red blood cells. When these features were identified by the ConvNeXt models, they marked a critical point in signalling an infection, which LLaMA translated into a layman’s understanding.

The use of natural language processing in combination with machine learning algorithms has its advantages in clinical settings as it helps make the results more understandable. This way, the system expands the original AI’s predictions with natural language descriptions to fulfil the requirements of healthcare workers. Extending the ability to justify the prediction made by the model helps enhance the credibility of the model’s predictions. It enables the clinician to act on the insights provided by the AI system more effectively. Ultimately, this integration makes the system more practically beneficial for diagnostic pipelines.

#### Deployment.

To develop the application, a Gradio interface with a well-organised and easily navigable layout was employed to facilitate the uploading of microscopic blood smear images with the subsequent immediate diagnostic output. The interface design prioritised usability, ensuring accessibility even for individuals with minimal technical expertise. When an image is uploaded, the system processes the image through a pre-processing, classification, and explanation phase. The results are presented to the user in both textual and graphical formats. It also builds on the features of Gradio’s interface that enhance the interaction, especially for individuals who may not be quite conversant with the software. After the image has been uploaded, the fine-tuned ConvNeXt models are applied to the image, and the output is either parasitised or uninfected. In addition, a LIME model visualises the image regions that led to the classification decision.

The additional textual explanation that LLaMA can help practitioners understand the outcomes more straightforwardly. This dual output, which is both visual and textual, significantly improves the understandability of the AI-based differential diagnosis. For this reason, the app has a supporting backend script that controls memory usage, especially in environments with constrained resources. It is the same whether the code runs on a GPU for fast computations or a CPU in less powerful environments; the app backend is designed to be non-memory bound.

This design helps the app work effectively even in regions with limited resources, which is typical for malaria-endemic areas. Since the app was developed based on Gradio, it has a high level of adaptability. It can be adapted for mobile versions or integrated into existing healthcare systems. This portability is especially valuable in malaria-endemic areas where portable diagnostic tools are crucial for front-line healthcare providers. With this app accessible online, installed on mobile devices or in healthcare systems of a specific region, the lack of time and AI in detecting malaria becomes relative, thus increasing the chances of curing patients in areas where malaria still needs to be solved.

## 4. Results

In this study, deep learning has been widely used for malaria parasite detection, and many evaluation metrics have been used to assess the performance of the models in each of the classification tasks. To evaluate the performance of the selected models such as Swin Tiny, ResNet18, ResNet50, ConvNeXt Tiny, ConvNeXt V2 Tiny and a re-modified version of ConvNeXt V2 Tiny, various criteria were used. These metrics are accuracy, precision, recall, F1 score and ROC-AUC (Receiver Operating Characteristic – Area Under the Curve). Relative measures, including log loss, MCC, specificity, balanced accuracy, Cohen’s kappa, G-mean, FPR and FNR, were also used to assess the discriminative ability of the models between parasitised and uninfected blood smears.

The basic measure is accuracy, in Equation 11, and is calculated as the number of correctly classified observations divided by the total observations. Mathematically, accuracy can be expressed as:


$$Accuracy=\frac{TP+TN\;}{TP+TN+FP+FN}$$ (11)

The symbols used are TP for True Positives, TN for True Negatives, FP for False Positives and FN for False Negatives. Since the dataset used in this study was balanced, accuracy is a good measure to use, although it may not be the best measure for imbalanced data. Both precision and recall, thus, give a more detailed picture.

Precision or Positive Predictive Value, shown in Equation 12, is the probability that a positive identification is correct. It is calculated as:


$$Precision=\frac{TP}{TP+FP}$$ (12)

Precision is crucial when false positives are to be avoided, for example, in a diagnosis where an incorrect result could subject a patient to unnecessary treatment.

Recall, or Sensitivity, is the ability to find all the positives out of the cases that are in fact positive. It is calculated as:


$$Recall=\frac{TP}{TP+FN}$$ (13)

A high recall rate, calculated as shown in Equation 13, means that the model accurately predicts most of the positives, which is especially important for tasks that involve risk, such as diagnosing malaria, when failure to identify a positive case (false negative) may have severe outcomes.

The F1 score, in Equation 14, integrates precision and recall into a single score that balances the trade-offs between precision and recall equally. The F1 score is defined as the harmonic mean of precision and recall:


$$F1=2\times \frac{Precision\;\times \;Recall}{Precision+Recall}$$ (14)

This metric gives us a more holistic picture of the model’s performance when it needs to balance trade-offs between precision and recall.

The ROC-AUC was also considered in this study, as shown in Equation 15. The true positive rate (TPR) is plotted against the false positive rate (FPR), giving the ROC curve, which graphically represents how the model is discriminatory. This performance is summarised by the AUC (Area Under the Curve) where the AUC score of 1 represents perfect discrimination and of 0.5 represents no discrimination. The AUC can be mathematically expressed as:


$$ROC-AUC=\int_{0}^{1}TPR\left(FPR\right)d\left(FPR\right)$$ (15)

A particularly useful metric for model performance in medical diagnostics is ROC AUC, whose purpose is to evaluate model performance across different thresholds and, thereby, across a range of decision boundaries.

Furthermore, uncertainty of predictions was measured using log loss, in Equation 16. This metric is a rigorous metric because it penalises overconfident but incorrect predictions more heavily than less confident ones. Mathematically, it is defined as:


$$Log\;loss=-\frac{1}{N}\sum_{i=1}^{N}[y_{i}\log\left(p_{i}\right)+\left(1-y_{i}\right)log(1-p_{i})]$$ (16)

where $y_{i}$ is the actual label and $p_{i}$ is the predicted probability for the positive class.

Quality of binary classifications was measured using Matthews correlation coefficient (referred to as MCC), given as Equation 17. It covers all four confusion matrix categories (TP, TN, FP, FN) and works well in imbalanced dataset cases, even though, the dataset used in this particular case was balanced. MCC is calculated as:


$$MCC=\frac{TP\times TN-FP\times FN}{\sqrt{\left(TP+FP\right)\left(TN+FP\right)\left(TP+FN\right)}}$$ (17)

True Negative Rate, OR Specificity, is the number of actual negatives identified as negatives. Recall addresses positive cases, and it is complemented by it. Agreement between the model’s predictions and true labels were also measured using Cohen’s kappa, refined by chance, and G-mean that is a geometric mean of sensitivity and specificity.

Moreover, using both False Positive Rate (FPR), in Equation 18, and False Negative Rate (FNR), in Equation 19, gives additional information about what type of errors the models made. FPR, calculated as:


$$FPR=\frac{FP}{FP+TN}$$ (18)

indicates how often the model incorrectly labels uninfected images as parasitised, whereas FNR, calculated as:


$$FNR=\frac{FN}{FN+TP}$$ (19)

reflects how often the model fails to detect actual parasitic infections.

Every metric has its advantages and disadvantages. For instance, while accuracy is easy to understand and calculate, it can be misleading with imbalanced datasets, though that was not an issue in the dataset used in this study [134,135]. Precision is relevant in situations with the least tolerance for error in predictions. At the same time, recall is important in situations requiring detecting as many positive cases as possible. The advantage of the F1 score is that it is a balanced metric, but it can be less useful when precision and recall are much different. ROC-AUC is a robust measure in different thresholds. However, it can be sensitive to the presence of imbalanced data; this was not a problem in this work. The log loss ensures a model is not overconfident in its predictions, and MCC provides a more complementary score for performance, especially when dealing with imbalanced datasets. FNR and G-mean increase the understanding of the model’s failure modes. Collectively, these measures demonstrate models’ capacity to minimise false negatives and false positives. Table 5 present model performance comparisons across these critical metrics.

**Table 5:** Model performance comparison across critical metrics.

Model	Accuracy	Precision	Recall	F1 Score	ROC-AUC
Swin Tiny	0.613988	0.572842	0.896420	0.699000	0.679830
ResNet18	0.625687	0.572930	0.987379	0.725112	0.839727
ResNet50	0.813671	0.731378	0.991502	0.841803	0.951111
ConvNeXt Tiny	0.958646	0.944025	0.975110	0.959316	0.991124
ConvNeXt V2 Tiny	0.544330	0.523228	0.998578	0.686663	0.815418
ConvNeXt V2 Tiny Remod	0.981249	0.979453	0.983123	0.981285	0.996633
**Model**	**Log Loss**	**MCC**	**Specificity**	**Balanced Accuracy**	
Swin Tiny	1.402099	0.276272	0.331555	0.613988	
ResNet18	1.681094	0.364074	0.263995	0.625687	
ResNet50	0.831209	0.671230	0.635839	0.813671	
ConvNeXt Tiny	0.116142	0.917790	0.942181	0.958646	
ConvNeXt V2 Tiny	2.224143	0.212159	0.090081	0.544330	
ConvNeXt V2 Tiny Remod	0.099783	0.962506	0.979376	0.981249	
**Model**	**Cohen’s Kappa**	**G-Mean**	**FPR**	**FNR**	
Swin Tiny	0.227975	0.545172	0.668445	0.103580	
ResNet18	0.251374	0.510552	0.736005	0.012621	
ResNet50	0.627341	0.794000	0.364161	0.008498	
ConvNeXt Tiny	0.917292	0.958505	0.057819	0.024890	
ConvNeXt V2 Tiny	0.088659	0.299922	0.909919	0.001422	
ConvNeXt V2 Tiny Remod	0.962499	0.981248	0.020624	0.016877	

The ConvNeXt models, especially the ConvNeXt V2 Tiny Remod, were highly able to differentiate between parasitised and uninfected samples with an accuracy of 98.1%, as shown in Table 5. This means the model can differentiate between malaria and other samples with high accuracy and low chances of misclassification, making it suitable for real-world applications. However, in the case of the Swin Tiny model, the accuracy was relatively low, standing at 61.4%, significantly indicating some discrepancies in the classification when differentiating between the two types of samples, which may raise a question regarding its practicability for real-time diagnosis.

Another critical factor in measuring the models’ performance is their capability to minimise the number of false positives. Here, the accuracy of the ConvNeXt V2 Tiny Remod was impressive, with a precision of 97.9%, as shown in Table 5. This high precision shows that the model made only a few mistakes when identifying parasitised samples, thus reducing the possibility of unnecessary treatment for unparasitised ones. On the other hand, the precision of Swin Tiny stands at 57.3%, which is an indication of the model’s propensity to classify healthy samples as infected ones. Such high false positive readings can result in mistreating patients and unnecessary procedures in a clinical context.

The ability of the model to correctly predict actual malaria cases is also important, as measured by recall. ConvNeXt V2 Tiny Remod had a strong recall, achieving 98.3% in this task. A recall of 98.3% means that the model failed to identify only 1.7% of the parasitised cases, which shows that the model has high sensitivity in detecting malaria-infected blood smear image. Nevertheless, a higher recall of 89.6% was observed from Swin Tiny, which shows that the model correctly identified a large number of parasitised samples; however, it also had a low precision, meaning that Swin Tiny often misclassified uninfected samples as parasitised.

The F1 score, which incorporates the trade-off between precision and recall, also confirmed the superiority of ConvNeXt V2 Tiny Remod with a score of 98.1%. This balance shows the model’s general performance in the classification task; the model could classify most positive cases without classifying many as negative. On the same note, the F1 score of Swin Tiny at 69.9% indicates the model’s inability to balance between detecting true positives and minimising false positives ideally, thus being less suitable for the fine-tuning task of malaria detection.

The accuracy and precision of the ConvNeXt models are not the only factors that make them stand out. The ROC-AUC value of the model that quantifies how well the model can differentiate parasitised and uninfected samples was 0.996 for ConvNeXt V2 Tiny Remod. This high value points to the model being well-equipped to distinguish between the two categories. However, the ROC-AUC of 0.679 for Swin Tiny shows that it did not perform well in this regard, which reaffirms the previous observation that it was not quite adept at making the correct classifications.

In terms of confidence in the predictions of the model, the ConvNeXt V2 Tiny Remod was once more the best amongst the models, with a low log loss of 0.099. This low score suggests that the model made highly certain decisions, thus limiting the chances of misdiagnosis to the minimum. On the other end of the spectrum, with a log loss of 1.40, the Swin Tiny failed to distinguish between parasitised and uninfected image classes, thus playing into its subpar performance.

Additionally, ConvNeXt V2 Tiny Remod fared well in the MCC with a score of 0.962, which measures the overall performance of a model for both false positives and false negatives. This score enhances the credibility of the model in consistently producing accurate outcomes. Swin Tiny’s MCC was also relatively poor at 0.276, indicating that it was unreliable and had a high level of variability in classifying the samples. This was especially seen in the model’s specificity, which did not produce many false positive results. Based on the results, ConvNeXt V2 Tiny Remod had a specificity of 97.9%. Thus, it rarely offered false positive classifications of uninfected samples as parasitised, which is essential to avoid unnecessary treatments for patients who do not have the disease.

The sensitivity for Swin Tiny was 33.2%, a lower value compared to the other algorithms; this can be attributed to the model’s tendency to classify uninfected cells as parasitic, thus reducing the model’s usefulness in clinical diagnosis. Regarding balanced accuracy, that is, the mean of recall and specificity, ConvNeXt V2 Tiny Remod occupies the leading position with a score of 98.1%. This high value indicates the model’s good performance on both the positive and negative classes because it can distinguish between them and avoid over-prediction cases where it may erroneously predict the image as having parasites when it does not. This is because the balanced accuracy of Swin Tiny stands at a low 61.4%, which shows comprehensive inefficiency regarding balance.

The Cohen’s kappa scores of the ConvNeXt models also showed that the models made almost accurate classifications of the images. The ConvNeXt V2 Tiny Remod achieved an accuracy of 0.962, which shows a high correlation with the true labels; on the other hand, Swin Tiny, with an accuracy of 0.228, shows a low correlation and is in line with the earlier findings that it performed poorly.

Considering G-Mean, a measure combining recall and specificity, ConvNeXt V2 Tiny Remod is the best, with a value of 0.981. This implies that the model could distinguish between the parasitised images and, at the same time, minimise over-prediction. For instance, Swin Tiny, with a G-Mean of 0.545, failed to strike this balance, affecting its performance.

These findings indicate that the ConvNeXt models are substantially better than the others for detecting malaria parasites in microscopic images, especially the ConvNeXt V2 Tiny Remod model. Such models provided high accuracy and precision and had low error rates, thus showing indications of suitability for practical use. Conversely, Swin Tiny could have been stronger in several aspects, limiting its capability to address this classification problem.

**Fig 7:**
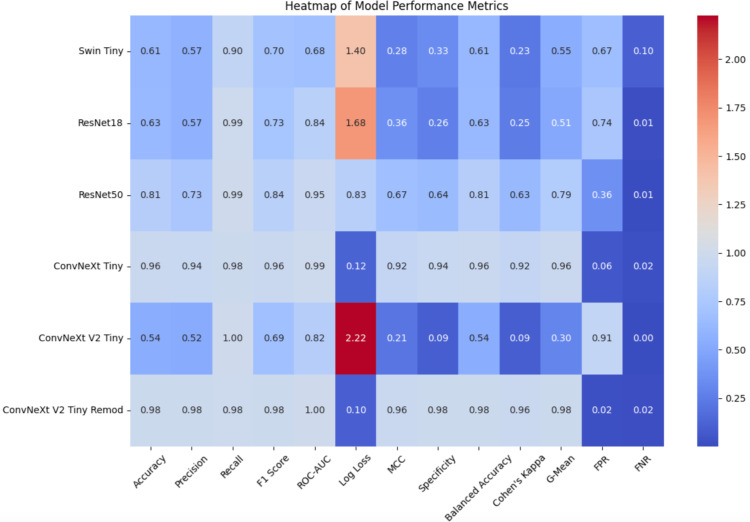
Heatmap of model performance metrics.

The heatmap in Fig 7 supports the tabulated results as they compare model performance across multiple metrics. Of all the models, ConvNeXt V2 Tiny Remod and ConvNeXt Tiny have the best accuracy, precision, recall, and F1 score, as well as low log loss, thereby proving to be efficient in identifying parasitised and uninfected blood smears. This is supported by their high specificity and moderate sensitivity, which enhances their performance. Conversely, Swin Tiny and ConvNeXt V2 Tiny models show less stable results with lower precision and higher false positive rates, thus weaker classification capabilities. The general comparison indicates that ConvNeXt V2 Tiny Remod is the most viable model for malaria identification.

This assessment further shows that the ConvNeXt models, especially the ConvNeXt V2 Tiny Remod, are more effective for malaria parasite detection in the blood smear slides. These models have high accuracy, precision, and balanced classification for medical imaging tasks and, therefore, have a great potential for enhancing diagnostic accuracy.

Even though the evaluation was done using the same augmented dataset, multiple safeguards were put in place to ensure the validity of this generalisation assessment. The pipeline for augmenting the data was applied stochastically to introduce diverse transformations over each training instance and prevent the potential of the model to overfit particular augmentation patterns. Model performance was rigorously evaluated through 5-fold cross-validation, giving evaluation capability over different data partitions. With consistently high accuracy (0.9812), F1 score (0.9813), ROC AUC (0.9966), and specificity (0.9794) as classification metrics, it is evident that the model generalises well on predictions beyond the augmented examples. The results suggest that by employing the augmentation strategy, the robustness of the model was enhanced without introducing systematic bias and the morphological integrity was not compromised.

The computational requirements and efficiencies of the evaluated models are summarised in Table 6, which includes training and evaluation times, speeds, and associated computational overheads.

**Table 6:** Computational requirements and efficiencies of the evaluated models.

Model	Training Time	Training Speed	Evaluation Time	Evaluation Speed	Overheads
Swin Transformer	~10 hours 13 minutes	~1:01:21 per epoch	~24 minutes 24 seconds	~3.23 samples/second	Hierarchical mechanisms enhance feature extraction but increase computational cost.
ResNet18	~3 hours 20 minutes	~20 minutes per epoch	~15 minutes 29 seconds	~5.10 samples/second	Lightweight architecture; minimal overhead, highly efficient in resource-constrained settings.
ResNet50	~7 hours 13 minutes	~43 minutes per epoch	~16 minutes 32 seconds	~4.77 samples/second	Increased depth leads to higher computation; balanced trade-off between speed and detail.
ConvNeXt Tiny	~25 hours 40 minutes	~2:34:00 per epoch	~24 minutes 15 seconds	~3.25 samples/second	Optimised for high-resolution feature mapping; moderate overhead due to deeper architecture.
ConvNeXt V2 Tiny	~30 hours 55 minutes	~3:05:00 per epoch	~29 minutes 18 seconds	~2.69 samples/second	Enhanced feature extraction increases computational load; well-suited for high-precision tasks.
ConvNeXt V2 Tiny Remod	~31 hours 10 minutes	~3:05:00 per epoch	~29 minutes 13 seconds	~2.70 samples/second	Customisation adds computational complexity but optimises diagnostic accuracy for resource-intensive tasks.

Different trade-offs between speed, efficiency and diagnostic accuracy are observed in the computational performance of the models. The most computationally efficient model was ResNet18, which trained in about three hours and evaluated at 5.10 samples per second. On the other hand, ConvNeXt V2 Tiny Remod trained in over 30 hours and evaluated much slower than ConvNeXt V2 Tiny. Nevertheless, it excels in high-resolution feature processing, and its increased diagnostic accuracy makes it an attractive choice for applications requiring high precision.

Swin Transformer and ResNet50 models, which balance computational demand and feature extraction performance, had moderate training and evaluation times while extracting robust features. In contrast, ConvNeXt Tiny and ConvNeXt V2 Tiny demonstrated a trade-off in which extra computational overheads enabled high-resolution feature mapping and generalisation power required for tasks that require complex diagnostic capabilities.

Table 7 displays the assessment outcomes of predictive performance combined with consistency metrics obtained from five-fold cross-validation executed on each model. The table displays average accuracy proportions, F1 score measurements and associated standard variations. Each model’s generalisability across different data splits is visible through these metrics, which serve as a foundation for evaluating the effectiveness of malaria parasite detection.

**Table 7:** Cross-validation results across five folds.

Model	Accuracy (mean ± std)	F1 Score (mean ± std)
Swin Tiny	0.6140 ± 0.0010	0.6990 ± 0.0004
ResNet18	0.6257 ± 0.0010	0.7251 ± 0.0008
ResNet50	0.8137 ± 0.0003	0.8418 ± 0.0001
ConvNeXt Tiny	0.9586 ± 0.0006	0.9593 ± 0.0006
ConvNeXt V2 Tiny	0.5443 ± 0.0001	0.6867 ± 0.0004
ConvNeXt V2 Tiny Remod	0.9812 ± 0.0003	0.9813 ± 0.0003

Paired t-tests were conducted to evaluate the statistical robustness of the differences in the observed performance of the tested models as measured by the accuracy scores obtained from 5-fold cross-validation. Thus, the performance of the ConvNeXt V2 Tiny Remod model was compared against each of the baseline models to see if the improvements were statistically significant.

As presented in Table 8, all pairwise comparisons were statistically significant (p < 0.00001), confirming that the performance gains of the ConvNeXt V2 Tiny Remod model are not due to random variation. These statistics favour the model’s better classification performance in malaria detection tasks.

**Table 8:** Paired t-test results (ConvNeXt V2 Tiny Remod vs others).

Comparison	t-statistic	p-value	Significant (p < 0.05)
ConvNeXt V2 Tiny Remod vs Swin Tiny	1973.8644	<0.00001	Yes
ConvNeXt V2 Tiny Remod vs ResNet18	2552.1977	<0.00001	Yes
ConvNeXt V2 Tiny Remod vs ResNet50	3745.4139	<0.00001	Yes
ConvNeXt V2 Tiny Remod vs ConvNeXt Tiny	221.8073	<0.00001	Yes
ConvNeXt V2 Tiny Remod vs ConvNeXt V2 Tiny	6178.6991	<0.00001	Yes

The paired t-test results validate that ConvNeXt V2 Tiny Remod shows better classification accuracy than all other five models (p < 0.00001 for every test). The ConvNeXt V2 Tiny Remod achieves both the highest mean accuracy rate of (98.12%) and F1-score rate of (98.13%) among all models and displays a small metric variability indicated by its standard deviation of 0.0003. These results further demonstrate robust consistency through the high generalisability of these models across different data partitions. However, these outcomes indicate that despite their reasonable performance, ConvNeXt Tiny and ResNet50, alongside other top-performing models, failed to surpass the model outputs from ConvNeXt V2 Tiny Remod. This demonstrates the effectiveness of label smoothing alongside AdamW and the data augmentation strategies in strengthening robustness within the model structure.

## 5. Discussion

This study shows that ConvNeXt architecture has many benefits and helps identify malaria parasites in thin blood smear images. The architectural design of ConvNeXt for high-resolution images allows it to have detailed local information and overall contextual information in the images. This two-fold function is beneficial in tasks involving basic and comprehensive information, such as medical diagnosis, since the images’ small details and overall picture must be analysed.

There are several reasons for the enhanced performance of ConvNeXt and, especially, the ConvNeXt V2 Tiny Remod model. First, the hierarchical feature extraction of the model can capture details of malaria parasites, including their shape, texture, and internal structure, while also capturing the general composition of the blood smear. This is very important in medical imaging, where diagnosis accuracy may depend on the finer details based on the image displayed.

Although this study’s dataset was from Bangladesh and thus restricts the geographic generalisability, data augmentation was applied to introduce variability. Future work should include datasets from other regions with different malaria strains to improve the model’s robustness. Collectively, the model could be expanded through global collaborations to improve its performance across different geographic contexts.

Another consideration for AI-based diagnostic systems is the possibility of bias arising from geographically or demographically limited training data. The dataset of this study mainly comprised blood smear images exclusively from Bangladesh. It raised issues with genetic diversity biases, such as ethnic differences in blood types and malaria strains. There is the potential that deploying the model in various regions or with different populations can affect its performance. Future studies should use more diverse datasets collected from different parts of the world, from different blood types, and different parasite strains to increase the generalisability of the model and ameliorate this bias.

Transfer learning was employed in the development of models in order to enhance their performance. The ImageNet dataset used to pre-train the models in this work provided them with a good starting point for general visual features such as edges, textures and shapes. This helped reduce the requirement for a large amount of annotated malaria data and improved the learning on the task-specific dataset.

To increase the model’s robustness to poor-quality images that are more likely to be encountered in low-resource settings, the model was trained and tested on images with noise, rotations, and varying brightness, using data augmentation techniques. Furthermore, using many data augmentation techniques increased the model’s generalisation capability. This enhanced the model’s stability and guaranteed that the model’s performance could be optimal in various practical situations.

However, these methods have some drawbacks which have been identified to affect their effectiveness. A limitation is that these models require access to computational resources, which may be scarce in some environments, particularly in resource-limited settings. Using architectures such as Swin-transformers and ConvNeXt can be computationally intensive, and this poses a challenge in practical applications where technology and infrastructure may be inadequate. On the other hand, access to these methods as an online service, where the computation can be deferred to reputable online platforms such as Amazon web services and Google Cloud, can be used as an alternative to enable access regardless of locally used computational resources [136–138].

While data augmentation and transfer learning could solve some problems, the problem of dataset representativeness still exists. The balanced dataset used in this study may not completely reflect the variability that can be potentially observed in real-world clinical settings, like staining technique, sample quality, or parasite life cycle stages. Additionally, the ConvNext models used in this study are the less advanced versions, ConvNext Tiny and ConvNext V2 Tiny models, due to the unavailability of computational resources. Future work can involve training more advanced models, such as ConvNext V2 XLarge, on more diverse and large datasets to find a way to generalise better.

The results from this study reveal the ability of ConvNeXt models, particularly in relation to the high recall and F1 scores that characterise these models and which are particularly useful for identifying malaria parasites. However, the utilisation of transfer learning and augmentation has drawbacks, including sensitivity to certain data distributions. This was somewhat offset by incorporating regularisation techniques such as the dropout.

Furthermore, including a new malaria diagnostic application within this framework emphasises the clinical significance of this study. The app builds upon ConvNeXt and incorporates methods such as LIME to facilitate real-time explainable AI diagnosis to healthcare professionals. This tool is especially useful in areas with a scarcity of specialists who are usually required to administer some of these tests. However, the app’s deployment might be restricted by the requirement for considerable computing ability and constant connectivity to the Internet, indicating more efficient field versions.

The ConvNeXt architecture shows clear clinical significance for malaria diagnosis in resource-limited environments. In high-burden areas, manual microscopy based on traditional methods is highly dependent upon skilled personnel and is prone to human error. An AI-based approach using ConvNeXt models to solve malaria detection has been proposed. It is automated and accurate in detecting malaria parasites and simultaneously tackles some of the key limitations of traditional approaches.

Additionally, the high accuracy and precision of ConvNeXt V2 Tiny Remod (98% accurate) demonstrate that deep learning-based systems are reliable for detecting malaria with accuracy similar to expert-level diagnosis. Such reliability may significantly alleviate the diagnostic workload of high-volume clinics and facilitate speeding up the diagnostic process, offering timely treatment and improving patient outcomes. Adding the layer of clinical significance to explainable AI techniques like LIME (for visual explanations) allows healthcare professionals to understand the AI model’s decision-making process. This puts into place an interpretability which builds trust with clinicians so that system outputs are both accurate and transparent.

When comparing this study with prior works, such as [15] those on RBCNet, key differences in the methods used are highlighted. In their work, Kassim et al. put forth a dual deep-learning pipeline by combining U-Net and Faster R-CNN to tackle the segmentation and detection of red blood cells in large, dense images. RBCNet was designed to achieve segmentation accuracy with a connected components approach and for dense cell environments with overlapping cells. In contrast, this study leverages ConvNeXt and other transformer-based models (such as Swin Transformer) for their accuracy and generalisability to detect malaria parasites.

One distinguishing feature is the dataset size and scope. [15] used a dataset of 965 images from 193 patients, with extensive cell-level annotations mainly in the context of red blood cell detection and segmentation. On the other hand, this study used Augmented Malaria Dataset for parasite classification, aiming at a wider goal to distinguish if a cell is infected or not. Augmented variations in the dataset of this study simulate real world conditions, such as noise and brightness changes, and are also consistent with the goal of improving model robustness for various settings. From a methodological standpoint, RBCNet had handcrafted dual-stage architectures tuned for specific challenges, such as cell clumping and dense overlap. In contrast, this study utilised pre-trained architectures like ConvNeXt, which use hierarchical feature learning and contextual awareness. The ConvNeXt V2 Tiny Remod outperformed the RBCNet segmentation-focused goals with an accuracy of 98%.

Another key advancement of this study is the integration of explainable AI (XAI) methods such as LIME and Llama 3.1, and the use of lightweight deployment models suitable for resource-constrained environments. These improvements solve the real-world hurdles of bringing AI tools into actual healthcare environments, including trust with healthcare professionals and computational constraints. In contrast [15], focused on computational efficiency and scalability in a diagnostic pipeline. Both studies emphasise the need to consider dataset diversity. As in this study, Kassim et al. noted that their dataset can be subject to bias because of geographic restrictions. Future efforts in both lines of research must focus on using diverse, globally sourced datasets to drive model generalisability and fairness.

Although the RBCNet [15] pushes segmentation-specific methods to dense imaging conditions, this work extends to real-world diagnostic applications with state-of-the-art architectures such as ConvNeXt to demonstrate clinical robustness, explainability, and adaptability.

This study further outlines computational requirements for malaria detection using deep learning models and provides valuable insights into trade-offs between efficiency, speed and diagnostic performance. The highest computational efficiency was achieved by ResNet18, which required minimal training and evaluation time. It is particularly well suited for deployment in resource-constrained environments, particularly where access to high-end computational infrastructure may be constrained. On the other hand, ConvNeXt V2 Tiny Remod models with more advanced architectural designs than others were designed for high-precision tasks and required more computational resources. This model required the longest training time of about 31 hours for 10 epochs of training and the slowest evaluation speed. Due to its ability to process high-resolution features and improve diagnostic accuracy, it is suitable for applications that require precise and reliable results, e.g., in clinical laboratories or centralised diagnostic facilities.

ConvNeXt Tiny and ConvNeXt V2 Tiny models demonstrated their ability to generalise and handle high-resolution feature mapping. However, their computational overheads, especially in training time, indicate that they require appropriate computational resources for practical deployment. These results corroborate the objectives of this study as they address the diverse needs of real-world applications. Lightweight architectures such as ResNet18 are feasible for resource-limited settings, maintaining diagnostic performance while being accessible and efficient. At the same time, high-performance models such as ConvNeXt V2 Tiny Remod demonstrate the adaptability of AI-based diagnostic systems to different deployment conditions and can be utilised for precision-oriented tasks.

Future work should improve these models for lower computational requirements while maintaining high diagnostic accuracy. Moreover, alternative deployment methods, including cloud-based services, can help alleviate local computational constraints and extend access to more sophisticated diagnostic tools. The considerations in these studies highlight the importance of balancing computational demands and diagnostic performance to realise the greatest impact of AI-based malaria detection systems in heterogeneous healthcare settings.

This study has important implications for malaria-endemic health systems with limited access to skilled personnel and advanced diagnostic facilities. The results suggest that ConvNeXt based models could be deployed to achieve accurate, automated malaria diagnosis using minimal resources. The models are adaptable to different imaging conditions through data augmentation and transfer learning and, thus very suitable for real-world use in under-resourced settings. The ConvNeXt V2 Tiny Remod model’s ability to achieve 98.1% accuracy means that it can help to decrease the reliance on manual microscopy, which is susceptible to human error and takes a lot of time and expertise. Direct implications of this are for reducing diagnostic delays and improving treatment outcomes in settings where early intervention is key to patient survival. Moreover, the study showcases the scalability of AI-driven diagnostics. An explainability framework is included that integrates LIME and LLaMA to make the system interpretable to healthcare professionals. The need for this transparency is critical to cultivating trust in AI-based systems and their adoption in clinical workflows.

This study did not include additional external testing data but rigorously managed external validity through methodological approaches. A methodology using 5-fold cross-validation helped prevent overfitting by validating every sample at least once per fold. A medically realistic augmentation pipeline executed simulations of different imaging conditions which appear during field diagnostic practice. The paired t-tests for statistical significance evaluation proved that the performance results remained constant across validation folds and reached extremely high statistical significance at p < 0.000001. The model demonstration shows universal application in a way that validates its general use even though no separate external validation dataset is available. In future works, further model validation will involve external datasets from different medical sources and geographical areas to assess its performance in realistic clinical settings.

## 6. Conclusion

Deep learning models, especially the ConvNeXt-based malaria detection models proposed in this study, are promising for real-world application, especially in low-resource settings. Malaria remains a major disease burden, especially in developing countries where there is a shortage of expert clinicians and well-equipped laboratories. The ConvNeXt V2 Tiny Remod model presented in this study with an accuracy of 98% presents an efficient method of automating malaria diagnosis. The scientific rationale is that ConvNeXt can be used to identify malaria parasites in blood smears because the architecture enables the model to capture detailed and contextual information as a microscopist would while examining blood smears under a microscope. This makes it very sensitive and specific in detection, which is vital in the early diagnosis and correct identification of the parasites.

Moreover, data augmentation and transfer learning are implemented in the model, which improves the model’s performance regardless of the imaging conditions. These models could help decrease diagnostic errors, reduce the time to diagnosis, and enhance the clinical management of malaria patients. The use of diagnostic services in mobile or edge devices also presents a way of extending the services to regions without access to such services, thus contributing to efforts to fight this global disease burden.

This system is scalable and interpretable and promises to be useful for integrating into national malaria control programs. Its ability to operate efficiently in challenging environments makes it a potential solution to enhance access to diagnostic services in low-income and remote areas. Future work may include integrating this system with mobile health platforms to increase accessibility further. Moreover, it could be applied to other parasitic diseases and provide a scalable framework for AI-driven diagnostics in global health.

The findings of this study show that ConvNeXt models, when optimally trained with varied data through data augmentation, using pre-trained models through transfer learning, can significantly enhance malaria diagnostics. This outcome holds a promise for deploying AI-based systems in rural healthcare facilities with limited access to diagnostic expertise. Future will focus on integrating this solution into mobile diagnostic platforms and validating performance across diverse geographic regions to enable further generalisability of the results.
